# Ostwald Process Intensification by Catalytic Oxidation of Nitric Oxide

**DOI:** 10.1021/acsomega.4c09111

**Published:** 2025-01-09

**Authors:** Jithin Gopakumar, Rune Myrstad, Rebecka Bo̷rresen Anda, Halvor Øien, Bjo̷rn Christian Enger, David Waller, Magnus Ro̷nning

**Affiliations:** † Department of Chemical Engineering, Norwegian University of Science and Technology (NTNU), Sem Sælands vei 4, NO-7491 Trondheim, Norway; ‡ SINTEF Industry, Kinetic, and Catalysis Group, P.O. Box 4760 Torgarden, NO-7465 Trondheim, Norway; ¶ YARA Technology Center, Hero̷ya Forskningspark, Bygg 92, Hydrovegen 67, NO-3936 Porsgrunn, Norway

## Abstract

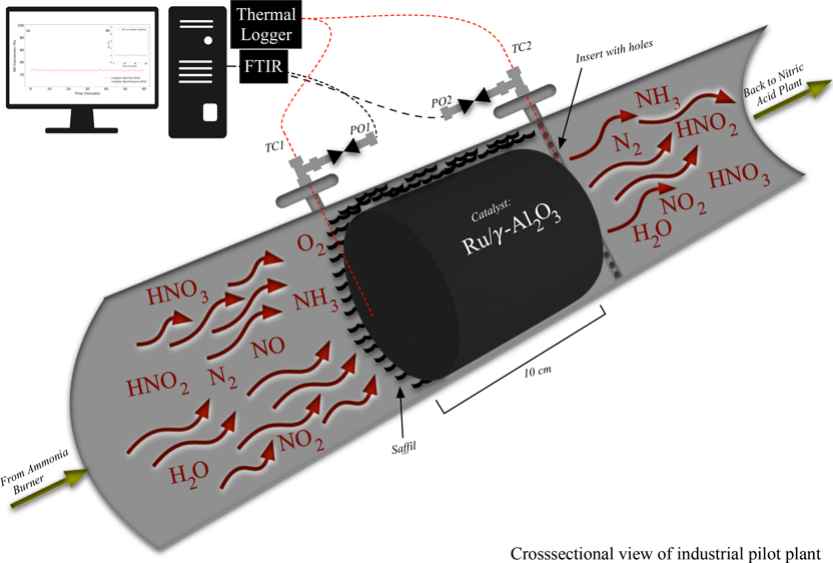

The Ostwald process is one of the commercial pathways for the production of nitric acid (HNO_3_), a key component in the production of nitrate fertilizers. The Ostwald process is a mature, extensively studied, and highly optimized process, and there is still room for further intensification. The process can be further intensified by catalyzing the homogeneous oxidation of nitric oxide to nitrogen dioxide. In this work, we explore the NO to NO_2_ oxidation capacity of ruthenium on γ-Al_2_O_3_ support wash-coated on to a cordierite monolith. In a lab-scale setup with simulated feed comprising 10% NO, 6% O_2_, 15% H_2_O and rest Ar, and 8% NO, 2% NO_2_ 5% O_2_, 15% H_2_O and rest Ar, the ruthenium and γ-Al_2_O_3_ wash-coated monoliths attained a steady conversion of 72 and 56%, respectively. A remarkable steady conversion of 20% more than the gas phase for 65 h over 4 days was presented by the Ru_Wc,γ–Al_2_O_3_,Cordierite_ catalyst in one of the pilot plants at Yara, a Norwegian fertilizer company. The results presented in this work clearly provide evidence to support the idea that ruthenium on a gamma-alumina catalyst support can oxidize NO to NO_2_ under industrial nitric acid production conditions and mark an important step in the intensification of the Ostwald process.

## 1 Introduction

Fertilizers play a vital role in modern agriculture, supporting global food production and ensuring food security for a growing population. At present, there are many different types of fertilizers; however, they are all primarily comprised macronutrients such as phosphorus, potassium, and nitrogen. Nitrogen is the rate-limiting nutrient for plant growth, and the production of nitrogen/nitrate-based fertilizers has always been a critical global challenge.^1^ The global demand for nitrate fertilizers is on a steady rise, closely linked to the ever-increasing global population and the need to meet their food needs.

Nitric acid is a corrosive mineral acid, mainly used to produce nitrate fertilizers, an essential that dramatically improves agricultural production in modern agrarian systems.^2,3^ Nitric acid also finds its applications in the production of paint, nylon, explosives, foam, etc. In 1908, the first nitric acid plant was constructed and used a method created by Wilhelm Ostwald and Eberhard Brauer in 1900–1901.^4^ The coke oven plant in Gerthe, Westphalia, produced ammonia gas, which was then mixed with air to produce nitrous fumes and absorbed in water to produce nitric acid. The Ostwald process is currently the cornerstone of modern industrial nitric acid plants.^3,4^ The Ostwald process can be summarized as eq 1:
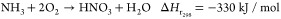
1This can be further subdivided into:^5,6^

- Ammonia (NH_3_) oxidation:
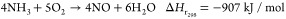
2
- Nitric oxide (NO) oxidation:
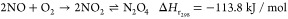
3
- Nitrogen dioxide (NO_2_) absorption:
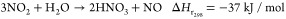
4

Commercially, nitric oxide oxidation happens in two ways, either cooling the gas after the ammonia combustor and/or providing sufficient residence time for homogeneous oxidation to NO_2_. As you can observe from the eqs 2–4, all of the steps in the Ostwald process are exothermic, and only a fraction of this energy is recovered.^7^

The oxidation reaction of NO to NO_2_ is one of the few known third-order reactions with a special quirk. NO is a free radical with an unpaired electron and, according to Honti,^5^ it oxidizes in two ways. The first step is an instantaneous dimerization reaction of NO with an equilibrium constant (*K*_NO_Dimer__) that increases with temperature as any other exothermic reaction. The second step is dimer oxidation by the consumption of oxygen to form NO_2_. If the overall rate of the NO oxidation reaction depends on the rate of the reaction of the second step and *K*_NO_Dimer__ increases with temperature, the overall rate of the NO oxidation reaction **r** is decreasing, giving rise to an inverse Arrhenius behavior. **r** is expressed as
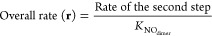
5The commercial producers take advantage of this inverse Arrhenius behavior to shift the equilibrium toward NO_2_ using heat exchangers. Here, a fraction of the high-quality heat is recovered. However, the degree of oxidation achieved is only 20%, and equilibrium predicts 55–90% between 400 and 300 °C and 4 bar(g) pressure (see Figure 8).^7^ This indicates that there exists an untapped potential for further oxidation of NO to NO_2_ and that an increase in the degree of NO oxidation at 400–300 °C and 4 bar(g) pressure could potentially recover additional heat of reaction to produce higher pressure and higher quality heat.

The main motivation of the work is to find a catalyst that can attain NO-NO_2_ equilibrium in the temperature range of 400–300 °C and 4 bar(g) pressure under industrial nitric acid production conditions. Therefore, (i) enables significant process intensification of nitric acid plants by high-quality heat recovery, (ii) reduces the industrial footprint, and (iii) decreases capital expenditure (CAPEX).^7^

Grande et al.^7^ used a Pt on γ-Al_2_O_3_ catalyst to study the kinetics of NO to NO_2_ under industrial conditions and found that the Ostwald process can be intensified by 10% with a stable heterogeneous catalyst that can oxidize NO in the temperature range of 250–350 °C at 4–5 bar(a). There have been efforts to replace the homogeneous NO oxidation at industrial conditions with a catalytic bed; however, to date, commercially, this process is carried out homogeneously with sufficient residence time, low temperatures, and high pressure.

Catalytic nitric oxide oxidation is not an uncharted research territory, it has been thoroughly investigated under lean conditions on various noble metals, base metals, metal oxides, perovskites, etc., where the feed composition consists of 0.0001–1% nitric oxide.^8−15^ Designing a catalyst to oxidize NO is challenging, it mainly faces two challenges, (i) gas phase conversion of NO to NO_2_, which means that the oxygen available for the catalytic reaction becomes limited in the feed, and (ii) the presence of strong oxidizers in the feed (NO, O_2_, NO_2_, HNO_2_, and HNO_3_), indicating oxidation of your catalyst or metal leaching. A critical parameter for designing catalysts that can oxidize NO in industrial plants is understanding the exact location of the catalytic reactor. For example, if the catalytic bed is closer to the exit of the ammonia combustor (eq 2), the lower NO_2_, higher O_2,_ and temperature favor catalytic activity and heat recovery compared to the reactor being close to the absorption column (eq 4). However, if the thermodynamics permits a higher conversion of NO at low temperatures and significant high-quality heat recovery is achieved, as suggested by Grande et al.^7^ Thus, the optimal catalyst bed operating temperature is a trade-off between the catalyst activity and the thermodynamics of NO oxidation.

A few early patents discuss the catalytic oxidation of NO.^16−18^ In addition to patents, Grande et al.,^7^ and our previous publications discuss NO oxidation under conditions relevant to nitric acid production.^19−23^

After the ammonia combustor, a typical industrial feed is composed of 10% NO, 6% O_2_, 15% H_2_O and equilibrium inert, which is corrosive and makes the analysis of the results even more challenging. Compared to base metals, noble metals can resist oxidation in certain acidic environments.^24^ Platinum (Pt) is a noble metal that has been studied extensively for its activity in the oxidation of NO to NO_2_.^14,19,25^ Due to the formation of platinum oxide under strongly oxidizing conditions, the stability of Pt catalysts for NO oxidation is in question.^26^

Ruthenium is a versatile noble metal with oxidation states from +8 to −2. As a result of this versatility, ruthenium is commonly used as a homogeneous catalyst but is also used in heterogeneous catalysis for various oxidation reactions, a popular catalyst for ammonia synthesis, and NO to NO_2_ oxidation at low concentrations of NO.^10,27−33^

Our earlier publications on NO oxidation activity in CeO_2_ support, a 5 wt % Ru on CeO_2_ catalyst was one of the most effective catalysts for attaining NO-NO_2_ equilibrium at industrial nitric acid production conditions.^23^ In another study, we closely monitored the performance of 0.5 wt % Ru on γ-Al_2_O_3_ catalyst which portrayed a redox behavior for the conversion of NO to NO_2_ with a promising low-temperature activity of 72% NO conversion at 340 °C and a pressure of 4 bar(g) under industrial nitric acid production conditions.

To the best of our knowledge, there has been no other research published than ours on supported Ru catalysts for NO oxidation at industrial nitric acid production conditions.^22^ This article reports on the industrial NO oxidation activity of a wash-coated Ru-γ-Al_2_O_3_ catalyst on a monolithic cordierite substrate. The corrosive nature of this process aggravates as you increase pressure to bridge the gap between a lab-scale setup and a pilot setup. With more NO_2_ produced and an increase in HNO_2_ and HNO_3_ concentrations in the feed is also observed. Another factor is the pressure drop across the reactor due to the shape and size of your catalysts, and monolithic catalysts are known to have a low pressure drop in reactors and thermal shock resistance.^34−36^ According to Harned and Montgomery,^37^ a monolithic substrate is better than a bead-shaped and Raschig rings substrate for an oxidizing system. According to Mouljin, Cybulski, and Strasser et al.,^38,39^ for commercial chlorination and oxychlorination reactions, mullite and cordierite were the two best substrates compared to conventional pellet catalysts, as they drastically decreased the pressure drop across the reactor, and the total heat integration improved. In our work, we decided to use a cordierite substrate for scale-up testing. Cordierite is insensitive to temperature changes and therefore has a near-zero thermal expansion coefficient.^38^ Because of their less porous surface, they are generally unsuitable as a support. However, they are widely used as substrates and in automotive industries, γ-Al_2_O_3_ is often deposited by wash coating due to its strong adhesion to cordiertie.^37,38^ In this work, we also explore the performance of ruthenium wash-coated monoliths at ambient and 4 bar(g) pressures in laboratory scale setups, and promising catalysts were scaled up to test in industrial pilots at Yara ASA. In addition, in situ XAS-XRD was used to investigate and characterize wash-coated ruthenium monoliths under conditions relevant to industrial nitric acid production.

## 2 Experimental Section

### 2.1 Catalyst Preparation

To prepare incipient wetness (dry) and wet-impregnated 5 wt % Ru on gamma alumina support catalysts (Ru_Dry,γ–Al_2_O_3__ and Ru_Wet,γ–Al_2_O_3__), a commercial γ-Al_2_O_3_ (BET surface area: 148 m^2^/g, size: 0–10 μm) support from Sasol Ltd. (CATALOX) was purchased. A solution (*S*_precursor_) was made by dissolving a calculated amount of RuCl_3_·*x*H_2_O (Sigma-Aldrich) in deionized water. Prior to incipient wetness (dry) impregnation of ruthenium on γ-Al_2_O_3_ support, the pore volume of the support was determined using N_2_ physisorption, and a calculated amount of metal-containing solution (*S*_precursor_) was added to the support. For the preparation of the wet-impregnated catalyst, a known weight of alumina support was mixed in an excess precursor solution (*S*_precursor_) and dried under ambient conditions overnight. Both dry and wet impregnated catalysts were further dried for 12 h at 120 °C and subsequently calcined in a flow of synthetic air (50 N cm^3^/min), heating at 5 °C/min from ambient to 400 °C, kept for 2 h, and subsequently cooled inside the calcination reactor. The calcined powder catalysts were pelletized, crushed, and sieved to 53–90 μm sieve fraction before reducing them in 5% H_2_/Ar as a function of temperature from ambient −400 °C with a heat rate of 5 °C/min.

For the preparation of monolithic catalysts, cordierite substrates (14.8 cm × 14.8 cm × 10 cm) were purchased commercially from CORNING (Celcor Substrates, 400 cells/in.^2^). Cordierite was cut into four pieces, each with a dimension of 7.2 mm × 7.2 mm × 5 cm (cuboidal shape) for testing in the NOO_*x*_ setup (see Section 2.3.1). A slurry (S1) of 35 wt % γ-Al_2_O_3_ catalyst powder was prepared in deionized water. Another solution (S2) was prepared using 30 wt % ruthenium precursor (RuCl_3_·*x*H_2_O, Sigma-Aldrich) in deionized water. To coat the monolith, the following steps are followed:

1. The cut monolith was dipped into S1 for 5 min and dried in air for 2 h at 120 °C.
2. The increase in weight was calculated, and step 1 was repeated until a coating of 30 wt % was achieved.
3. After achieving a coating of 30 wt %, the monolith of the support substrate was dried at 500 °C overnight.
4. The dried support substrate was immersed in S2 on one of the longer ends of the monolith for 10 min and on the other end for another 10 min (capillary forces act inside the monolith).
5. The surface of the same support substrate was spray-coated with S2 using a spray gun and pressurized Ar.
6. The coated monolith was dried in air for 12 h at 120 °C and calcined at 400 °C for 2 h and subsequently cooled inside the calcination reactor.
7. The calcined monolith was reduced in 5% H_2_/Ar as a function of temperature from 50 to 400 °C with a heat rate of 5 °C/min.
8. The reduced monolith was weighed, and the metallic ruthenium weight percent was calculated.
9. Steps 4–8 were repeated until sufficient ruthenium loading was achieved.

Wash coating of a monolith is very tricky, especially in the inner channels of the monolith. There may be areas where the gamma-alumina coat is absent and where ruthenium directly binds to the cordierite substrate. Therefore, to understand the activity and role of the cordierite substrate, ruthenium was also directly coated on cordierite following steps 4–8 to make the Ru_Wc,Cordierite_ catalyst (Wc stands for wash-coated). For the Yara tests in the NOO_*x*-Pilot_ setup, the same preparation method as above was used on a cylindrical cordierite substrate (*l* = 10 cm and OD = 7.4 cm). The monolith shape was changed for the tests in the NOO_*x*-Pilot_ setup to accommodate the amount of catalyst weight to match the linear velocity and the area of exposure. Details and designations of the catalysts used for the tests in the NOO_*x*_ setup are presented in Table 1.

**Table 1:** Catalyst Designations

catalyst^a^	details^b^	catalyst form
Ru_Dry,γ–Al_2_O_3__	5 wt % ruthenium dry impregnated on γ-Al_2_O_3_ support	Powder
Ru_Wet,γ–Al_2_O_3__	5 wt % ruthenium wet impregnated on γ-Al_2_O_3_ support	Powder
Ru_Wc,Cordierite_	5 wt % ruthenium wash-coated on cordierite directly^b^	Monolith
Ru_Wc,γ–Al_2_O_3_,Cordierite_	5 wt % ruthenium wash-coated on γ-Al_2_O_3_ coated cordierite^b^	Monolith

a All catalysts are prereduced in 5% H_2_/Ar as a function of temperature from 50 to 400 °C with a heat rate of 5 °C/min.

b The weight of the ruthenium coating is measured by weighing the monolith before and after reduction.

### 2.2 Catalyst Characterization

Brunauer–Emmett–Teller (BET) specific surface area was measured at liquid nitrogen temperature (−196 °C) for γ-Al_2_O_3_ support, Ru_Dry,γ–Al_2_O_3__ and Ru_Wet,γ–Al_2_O_3__ catalysts using N_2_ adsorption. 100 mg of each sample were degassed at 200 °C for 30 h on a VacPrep 061 degasser before transfer to the Micromeritics TriStar II 3020 surface area and porosity analyzer.

Dispersion measurements were recorded using the ASAP 2010S Micrometrics unit at room temperature for fresh and spent samples of the Ru_Dry,γ–Al_2_O_3__ and Ru_Wet,γ–Al_2_O_3__ catalysts. A sample of known weight was loaded into a U-shaped quartz reactor, and the bed temperature was controlled by using a thermocouple. Before chemisorption, the sample was dried at 100 °C for 30 min and reduced by hydrogen in a temperature increase of up to 400 °C with 5 °C/min ramp-rate and dwell time of 1 h. The isotherm was measured in the pressure range 150–400 mmHg. The dispersion was calculated on the basis of strongly adsorbed CO, and we assume an adsorption ratio of 1 for CO/Ru.^40,41^

Ex-situ X-ray diffractograms for catalyst samples and support were obtained using a Bruker D8 Advance X-ray diffractometer (D8 Davinci) at 40 kV and 40 mA, using the wavelength of Cu K_α_ radiation (1.54060 Å). The diffractograms were recorded in the 2θ range of 5–75° with a 0.1° slit opening.

In-situ X-ray absorption spectroscopy and X-ray diffraction (XAS-XRD) experiments at the ruthenium K edge (22.1172 keV) were carried out at the Swiss-Norwegian Beamlines (SNBL) BM31 at the European Synchrotron Radiation Facility (ESRF), France. Figure S2 presents the experimental setup used for in situ experiments. The reactor consists of a quartz capillary with internal diameters (ID) of 0.2 cm and a known amount of catalyst sample with wads of quartz wool at either end of the catalyst bed (bed length = 1 cm). The reactor was mounted on a custom stainless steel (SS-316) bracket and exposed to X-rays, the capillary temperature being controlled using a cartridge heater designed by Marshall et al.^42^ A dedicated setup with mass flow controllers was used to feed desired concentrations of NO, NO_2_, O_2_, and He (WHSV: 24,000 N cm^3^/g_cat_ h). Water was fed by using a liquid flow controller and an evaporator. A back pressure controller was used to control the pressure inside the capillary reactor for 4 bar(g) experiments. To reduce the homogeneous conversion of nitric oxide before the bed, a tube in tube of 1/32” was used to feed NO_*x*_, such that oxygen, water, and helium meet NO_*x*_ at the inlet of the catalyst bed. In-situ X-ray diffractograms were collected with a Pilatus detector (Dectris) using monochromatic radiation (λ = 0.25 Å). A NIST 660a LaB_6_ standard was used to correct for the detector distance, instrumental peak broadening, and wavelength calibration. The extended X-ray absorption fine structure (EXAFS) was measured at 50 °C in 100% He to analyze the local environment of ruthenium for fresh, reduced, and spent catalysts. X-ray absorption near-edge structure (XANES) profiles were recorded during the reduction temperature ramp and the isothermal NO oxidation run at 350 °C. The in situ XAS-XRD program is described in Figure 13 (i). The catalyst sample was first reduced in a temperature ramp from 50 to 400 °C (ramp rate of 5 °C/min) in 5% H_2_/He with 2 min dwell at 400 °C, before being subjected to isothermal NO oxidation at 350 °C. For monolithic catalysts (Ru_Wc,γ–Al_2_O_3_,Cordierite_ and Ru_Wc,Cordierite_ catalysts), an isothermal NO oxidation reaction was performed at 4 bar(g) with a sieve fraction of 150–200 μm and two feed conditions with 0.5% NO, 1% O_2_, 1.5% H_2_O and He balances at 350 °C or 0.8% NO, 0.2% NO_2_, 1% O_2_, 1.5% H_2_O and He balances. The powder catalysts Ru_Dry,γ–Al_2_O_3__ and Ru_Wet,γ–Al_2_O_3__ (sieve fraction: 53–90 μm) were subjected to isothermal NO oxidation reaction at ambient pressure with two feed conditions with 0.5% NO, 1.3% O_2_, 0.75% H_2_O and He balances at 350 °C or 0.25% NO, 0.25% NO_2_, 1.3% O_2_, 0.75% H_2_O and He balances. The differences in reaction conditions and pressure are mainly due to differences in the catalyst sieve fraction, catalyst weight, and realistic space velocity at the beamline. A lower concentration of NO and NO_2_ was used for the in situ XAS-XRD studies due to the safety restrictions at ESRF. Due to limitations in the flow ranges of mass flow controllers, compromises were made for the reaction space velocity. The ruthenium edge step of fresh and spent catalyst samples was closely monitored to confirm the absence of RuO_4_. As standards, the EXAFS of the ruthenium foil (Ru^0^) and RuO_2_ (Ru^4+^) powder (Sigma-Aldrich) were measured ex situ in transmission mode. Ex situ X-ray diffractograms of cordierite, metallic ruthenium powder, ruthenium oxide (RuO_2_), and γ-Al_2_O_3_ at 50 °C were also recorded for comparison. Additionally, Athena, part of the Demeter software package, was used to analyze all the XANES components collected and perform linear combination fitting (LCF) with Ru^0^ and RuO_2_ XANES as standards. All EXAFS were treated in Athena with a background subtraction factor (*R*_bkg_) of 1.1–1.3 AA.^43^

### 2.3 Experimental Setup for Catalytic Activity Measurements

#### 2.3.1 Lab Scale Setup (NOO_*x*_)

**Figure 1:**
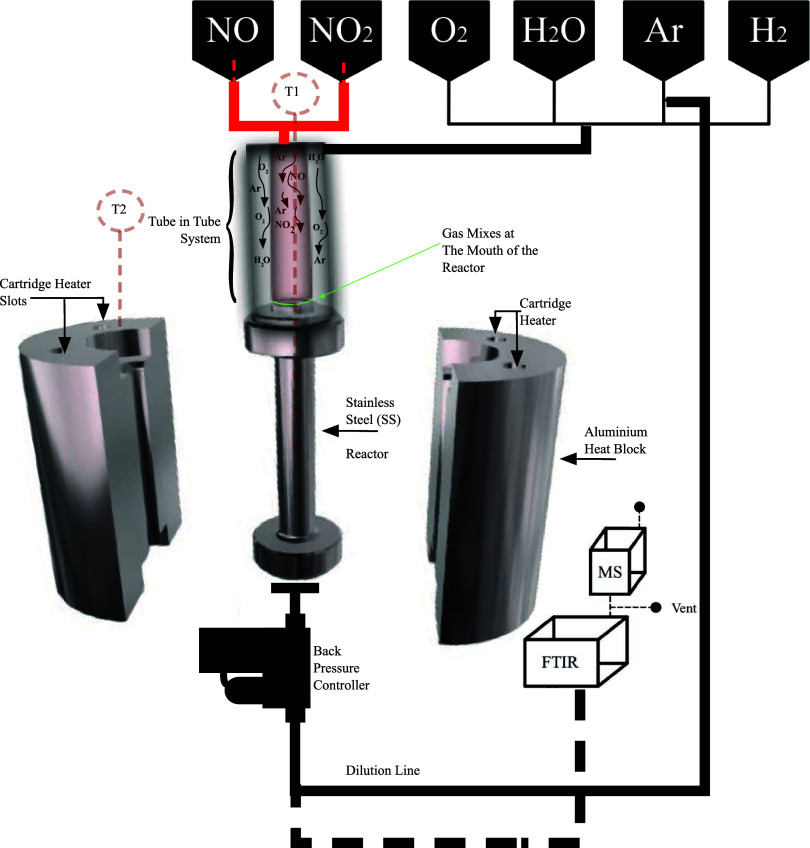
Process flow diagram with tube-in-tube reactor design for minimizing nitric oxide gas-phase conversion.

A sophisticated experimental setup (see Figure 1) was prepared for the oxidation of NO under industrial nitric acid production conditions. The experimental setup is briefly detailed in Section S2 and in our previous work.^19−23^ To summarize, the process lines (SS-316), valves, analyzers, gas detectors, and water feeders were placed inside a polycarbonate cabin connected to a corrosion-resistant ventilation unit. The reactant gases, NO, O_2_, H_2,_ and Ar were obtained from the commercial supplier Linde-Gass AS. NO_2_ during activity tests was produced in situ by homogeneous oxidation of nitric oxide and oxygen.^23^ Water vapor was controlled and fed using a controlled evaporator mixer (CEM) from Bronkhorst. All gas lines downstream of the mass flow controller to the reactor and after the reactor are preheated to 200 °C to ensure that there are no cold spots for condensation of the water. A high-precision back pressure regulator with an SS-316 diaphragm (ULHT Equilibar BPR) was introduced downstream of the reactor to carry out pressure experiments.

An infrared gas analyzer (MKS MultiGas 2030-HS FTIR Gas Analyzer, 5.11 m path length) is used to analyze the product stream and give direct composition for NO, NO_2_, N_2_O, H_2_O, NH_3_, HNO_2_, and HNO_3_ using precalibrated data obtained from MKS. A mass spectrometer (Pfeiffer Vacuum ThermoStar GSD 301 T3 Benchtop Mass Spectrometer) is used to monitor Ar and homonuclear diatomic molecules such as the concentrations of the aforementioned O_2_ and N_2_ in the product stream.

Catalyst performances were evaluated as a measure of conversion of NO to NO_2_ (%) with respect to temperature in the range of 150–400 °C in two different feeds; Feed (i) 10% NO, 6% O_2_, 15% H_2_O and rest Ar and feed (ii) 8% NO, 2% NO_2_, 5% O_2_, 15% H_2_O and rest Ar, with a space velocity of 24,000 N cm^3^/g_gcat_ h. Conversion of NO (%) is calculated as
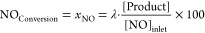
6
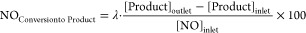
7
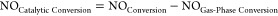
8where [NO]_inlet_ is the inlet concentration of NO and [Product]_inlet/outlet_ defines the inlet and outlet concentrations of the product in question. For example, if the product is NO_2_, conversion of NO to NO_2_ (%) is defined as ([NO_2_]_outlet_ – [NO_2_]_inlet_) × 100/[NO]_inlet_.

The inlet concentrations of any product ([Product]_inlet_) are measured during gas phase experiments. λ = 0.99, which accounts for the volume changes that arise from the reaction.^44^ NO_Gas-Phase Conversion_ was measured by performing blank tests. For a monolith, an uncoated cordierite substrate was subjected to the same conditions as the catalyst for gas-phase measurement. For a powdered catalyst, a mix of catalyst support and SiC is subjected to the same conditions as the catalyst for the gas-phase measurement.

Isothermal experiments at 350 °C and 4 bar(g) pressure were performed with feed (i): 10% NO, 6% O_2_, 15% H_2_O and rest Ar and feed (ii): 8% NO, 2% NO_2_, 5% O_2_, 15% H_2_O and rest Ar, at WHSV = 24,000 N cm^3^/g_gcat_ h. Prior to 4 bar(g) measurements, the monoliths were pretreated with 5% H_2_/Ar at 400 °C and ambient pressure. The reactor is pressurized to 4 bar(g) in 100% Ar at 350 °C.

#### 2.3.2 Pilot Scale Setup (NOO_*x*-Pilot_)

The NOO_*x*-Pilot_ setup was designed and operated at Yara, Porsgrunn, Norway, downstream of the FAL-2 (Forso̷ks Anlegg) pilot plan. Figures S3 and 2 present the FAL-2 pilot model and schematics. The representations clearly mark the boundary between the NOO_*x*-Pilot_ setup and the FAL-2 pilot plant. FAL-2 is a monopressure (4 bar(g)) nitric acid pilot plant.

**Figure 2:**
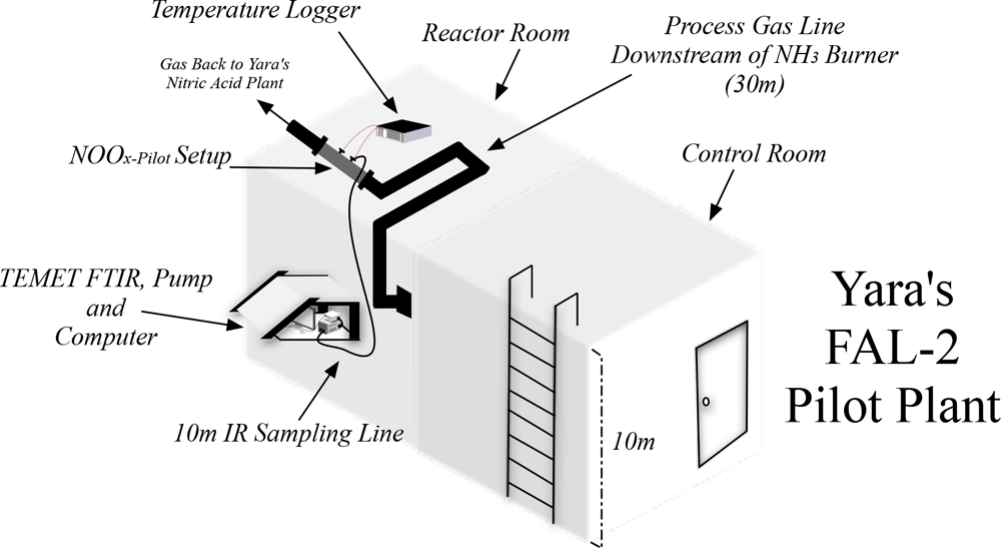
Model representation of the FAL-2 pilot at Yara, Porsgrunn, Norway.

A typical FAL-2 plant startup involves evaporating liquid ammonia obtained from one of Yara’s nitric acid plants and further mixing the evaporated ammonia with hot air. Before the mixture is introduced to the ammonia burner, the premixed gas will be filtered. The different zones of the ammonia burners are preheated to 200 °C, and once the temperature stabilizes in the burner, the middle zones (with the Pt–Rh gauze catalyst) are ignited to 950–960 °C. The NH_3_ concentration is controlled and maintained at 10–11% by controlling the flow of ammonia and air.

**Figure 3:**
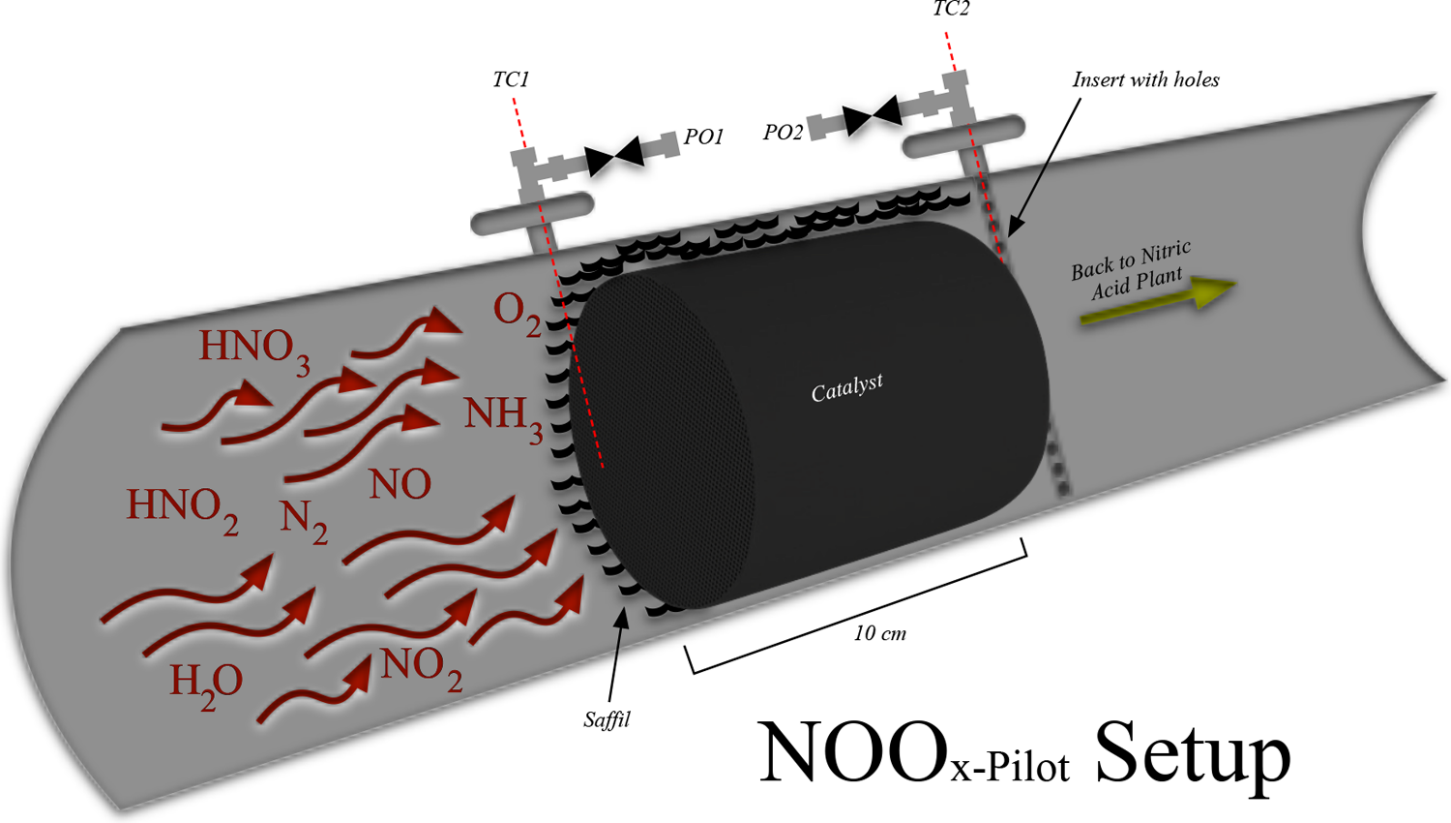
Cross-sectional representation of the NOO_*x*-Pilot_ setup at Yara, Porsgrunn, Norway.

The sample ports (PO1 and PO2) are directly connected to the TEMET FTIR TEMET Dx-1000 analyzer probe, and the thermocouples (TC1 and TC2) are connected to a temperature logger for measurements. Figure 3 presents the cross-sectional flow through the bed and the NOO_*x*-Pilot_ setup. To hold the catalyst bed between the PO1 and PO2 sampling ports, an insert (SS-316, 1/2 in.) of 83 mm was used. The inset was machined with holes to allow flow through the PO2 sampling port. Thermocouples TC1 and TC2 are placed in the center of the inlet and outlet of the monolith. Saffil is an alumina fiber that resists heat up to 1600 °C and was used to fill the gap between the lateral area of the monolith and the reactor as presented in Figure 3.

Figure S4 presents the NOO_*x*-Pilot_ setup scheme with a cropped image of the real setup at the Yara FAL-2 pilot plant rooftop. The pilot setup consists of one horizontal stainless steel reactor (SS-304) of 82.5 mm internal diameter, two samples, and temperature ports (at the inlet and outlet of the catalyst bed).

The process gas from Yara’s NH_3_ ammonia burner passes through the NOO_*x*-Pilot_ setup for measurements. The process gas leaving the ammonia burner typically consists of 10–11% NO_*x*_ and 15–20% water at 950–960 °C and 4 bar(g) pressure. Process temperature and flow rate were controlled by tuning the FAL-2 pilot located upstream of the NOO_*x*-Pilot_ setup (see Figure S3). There exists approximately 30 m of tubing between the exit of the ammonia burner and the NOO_*x*-Pilot_ setup. This gas line downstream of the ammonia burner is exposed to the atmosphere and is subject to natural cooling. Therefore, the process gas temperatures drop from 950 to 960 °C to 330–350 °C by the time it reaches the NOO_*x*-Pilot_ setup. During gas-phase and catalyst activity measurements, weather data was collected around the FAL-2 pilot plant to compare ambient cooling and activity loss.

Prior to activity measurements, catalyst monoliths were prereduced in 5% H_2_/N_2,_ and gas-phase measurements were also collected with an uncoated cordierite monolith. Gas sample ports (PO1 and PO2, see Figure S4) were placed before and after the catalyst bed to measure the composition of NO, NO_2_, H_2_O, and NH_3_ with an FTIR TEMET analyzer. The conversion of NO to NO_2_ was measured at a space velocity of 242–240 N m^3^/g_gcat_ h and a linear velocity of 12.47. The composition of the gas fluctuated throughout the measurement; however, a typical composition of 10–11% NO_*x*_, 3–6% O_2_, 15–20% H_2_O and rest of N_2_ was assumed as 10–11% NH_3_ was fed into the burner. The pilot tests met additional challenges, and several assumptions and measures were taken to prevent them. The following list describes the challenges and respective measures:

#### 2.3.3 Challenges and Measures

- The cost of the ruthenium precursor was the bottleneck for pilot testing in the NOO_*x*-Pilot_ setup. The normal space velocity of the pilot FAL-2 was 242–240 N m^3^/g_gcat_ h which is 10,000 times higher than what is used for operation in the NOO_*x*_ setup. Scaling a catalyst according to the space velocity is not ideal because the space velocity accounts for the flow rate over a volume of the catalyst. The area of the reactor and the shape of the catalyst were not taken into account in this case. Therefore, the linear velocity was used to scale the catalyst for experiments in the NOO_*x*-Pilot_ setup. However, because of the cost of the ruthenium precursor, a trade-off was made with respect to the size of the catalyst bed. The total weight of the catalyst coated on a monolith was 63 g, with a bed length of 10 cm.
- As mentioned above, the NOO_*x*-Pilot_ setup exists downstream of the ammonia burner (see Figure S3), and in reality, the location is on top of the FAL-2 pilot plant exposed to ambient conditions. Therefore, the catalyst change from blank tests includes shutting down the FAL-2 pilot plant after venting and cooling down the entire FAL-2 pilot plant and the NOO_*x*-Pilot_ setup with a high flow of air for 1–3 h. The FAL-2 pilot start procedure was also challenging for catalyst testing. The ammonia burner is slowly heated to 200 °C at 4 bar(g) pressure by controlling the flow of air and ammonia through the evaporator (located upstream of the burner as presented in Figure S3). Once the burner is at 200 °C, an ignition of H_2_ is used to get the burner up to 900–950 °C in a matter of seconds. During this ignition, the downstream of the burner will be at 60–100 °C, causing the formation of liquid nitric acid that wets the monolithic catalyst located in the NOO_*x*-Pilot_ setup. The ruthenium catalyst placed inside the NOO_*x*-Pilot_ setup does not withstand nitric acid at low temperatures, leading to the oxidation of the catalyst. Therefore, we assume that only a fraction of the total catalyst will be reduced, and the remainder will be oxidized.
- The temperature of the catalyst bed was continuously measured before and after the reactor through the TC1 and TC2 ports (see Figure S4). As previously mentioned, the gas temperatures of the process drop from 950 to 960 °C to 330–350 °C by the time it reaches the inlet of the NOO_*x*-Pilot_ setup, contributing to the gas phase conversion of NO to NO_2_. The temperature drop is mainly affected by the surrounding conditions (air temperature, rain, and/or snow) of the NOO_*x*-Pilot_ setup. Therefore, the weather data was continuously monitored and collected through the Norwegian klima service senter.^45^ Due to sudden changes in the inlet temperatures, the performance of the catalysts was evaluated as the degree of conversion compared to that of the gas phase. Conversion before or after the catalytic bed is calculated as
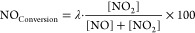
9The degree of catalytic conversion (%) in the NOO_*x*-Pilot_ setup is calculated as
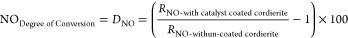
10where *R*_NO_ is calculated as the ratio of NO_Conversion_ at the exit (PO2) and entrance (PO1) of the bed.

An FTIR TEMET Dx-1000 analyzer is used to analyze the composition of NO and NO_2_. The analyzer consists of a probe, a pump, and 11 m of heated line. The lines to the analyzer were heated up to 180 °C, and the analyzer gas cell was heated to 190 °C. The IR requires a minimum flow of 3 L for analyzing the product stream. Other gases such as inert, H_2_O, HNO_3_, HNO_2,_ and O_2_ were not analyzed. The concentrations of NO and NO_2_ are calculated using precalibrated IR spectra. The calibrations for the reference gases were reacquired and resaved in Calcmet software for the TEMET FTIR.

### 2.4 Aspen Model for NOO_*x*-Pilot_ Gas-Phase Measurement

In the existing commercial plant, NO is oxidized to NO_2_ via a homogeneous gas-phase reaction in the piping and heat exchangers that are located downstream of the ammonia burner. Yara uses an Aspen Plus simulation to evaluate the degree of homogeneous oxidation by analyzing the temperature differences between two points in the process stream. We know that 10% NO_*x*_ is present after the ammonia burner since 10% NH_3_ is fully combusted; however, due to gas-phase oxidation and inaccuracy in industrial composition measurements (HNO_3_ formations), we assume a typical composition of NO and NO_2_ in the feed.

**Figure 4:**
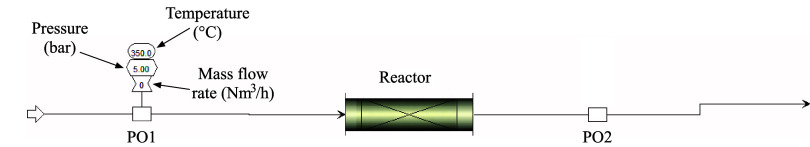
Aspen flow diagram used for simulating the NOO_*x*-Pilot_ gas-phase nitric oxide oxidation.

In this work, an Aspen Plus (Advanced System for Process Engineering) V12.1 was used to simulate the temperature increase and gas-phase NO to NO_2_ conversion (%) across a 10 cm bed with a process gas flow of 240 N m^3^/h and a feed composition of 7.4% NO, 2.6% NO_2_, 5% O_2_, 15% H_2_O, and balance nitrogen. This feed composition matches the results of measurements we took at the catalytic bed’s inlet via PO1 (presented in Figure 3) in the NOO_*x*-Pilot_ setup during gas-phase measurements. Figure 4 presents the flow diagram of the Aspen simulation. The simulation was developed by Yara, Porsgrunn, Norway, and is equipped with NO oxidation kinetics, where the forward reaction rate is expressed as^7^
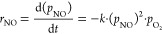
11The rate constant is given as
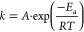
12and the reverse reaction rate is expressed as
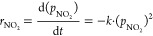
13Here, *p*_*x*_ (*x* = NO, NO_2,_ and O_2_) represents the partial pressure of species *x*, *k* represents the reaction rate constant, *R* is the ideal gas constant, and *T* represents temperature.^5^ The corresponding values for the activation energy (*E*_a_) and the pre-exponential factor (*A*) for the gas-phase reaction were taken from the literature.^5^

We know that the oxidation of nitric oxide has an inverse Arrhenius behavior, that is, with increasing temperature the rate declines. Therefore, the activation energy for the forward reaction is negative. The modeled reactions 11 and 13 represent the overall reaction and not the elementary steps. For gas-phase reaction simulation purposes, an RPlug reactor was used as it is the most suitable reactor to represent the NOO_*x*-Pilot_ setup, and only vapor phases were considered valid. The reactor dimensions were 10 cm in length and 82.5 mm in diameter. For the simulation, the temperature of 350 °C and the pressure of 4 bar(g) were used. In this simulation work, the feed and product process gas was considered to contain NO, NO_2_, H_2_O, O_2,_ and N_2_. Dinitrogen tetroxide, nitric, and nitrous acids were assumed to be absent and were not part of the simulation. A Soave–Redlich–Kwong equation of state was used in this work. The NO conversion (%) at each bed length is calculated by eq 9.

## 3 Results and Discussion

### 3.1 Gas-Phase Experiments

We have already discussed and established the inverse temperature dependence of nitric oxide oxidation. Industrial nitric acid plants utilize this trait to facilitate homogeneous oxidation of NO to NO_2_ and use a series of heat exchangers to remove heat and promote forward reaction. The following factors affect gas-phase conversion:

- Dead-volume
- Cold-spots (thermal gradients)
- Residence time
- Reactant concentration

#### 3.1.1 Experiments at NOO_*x*_ Setup

Transforming this bulky homogeneous NO to NO_2_ oxidation process to a catalytic reaction requires an efficient catalyst that is not inhibited by NO_2_ or H_2_O. However, obvious challenges exist in emulating this industrial process for any research work on the laboratory scale. Optimization of flow rate and residence time was carried out by Yara and Salman.^46^ The deciding factor for the optimal flow rate (200 N cm^3^/min) and residence time was a compromise between the operating costs and gas-phase conversion.

The process gas is mixed before the reactor in a tube-in-tube design (as presented in Figure 1), and after the reactor, the product gases are diluted using argon with a dilution ratio of 5–10 (argon flow/product gas flow). The thermal gradients before and after the reactors were minimized by heating the process lines to 200 °C, and the gradients in the reactor were minimized using SiC as a diluent.

**Figure 5:**
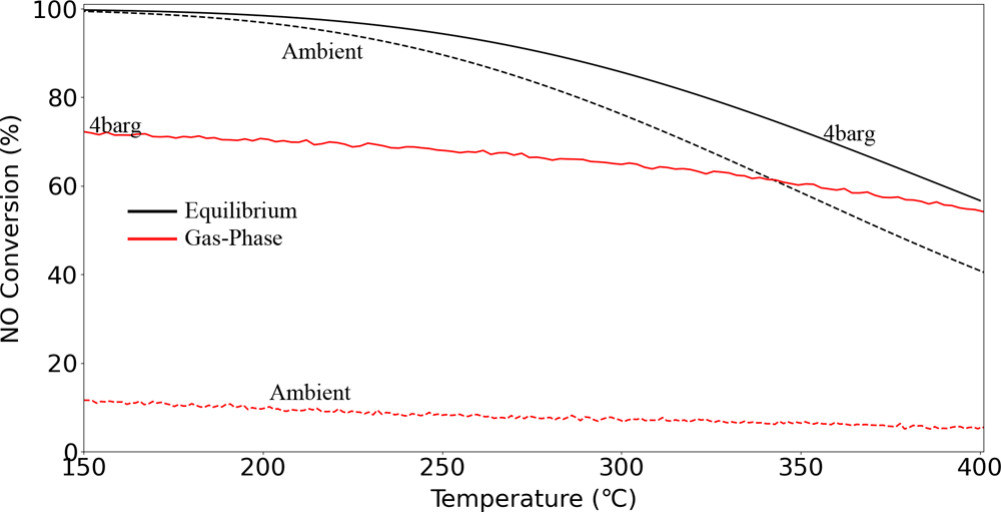
Gas-phase NO to NO_2_ conversion (%) for noncoated monolith catalyst substrate as a function of temperature in 10% NO, 6% O_2_, 15% H_2_O, and rest Ar with a heat rate of 5 °C/min at WHSV = 24,000 N cm^3^/g_gcat_ h at ambient pressure and 4 bar(g) pressure.

In this research work, we mainly used a cordierite monolith substrate. Figure 5 presents the gas-phase conversion for noncoated monolith catalyst substrates as a function of temperature in wet conditions. Elevated gas-phase conversion at 4 bar(g) pressure underscores the importance of minimizing gas-phase conversion. The difference between gas-phase conversion and equilibrium at 350 °C is only 12% for 4 bar(g) experiments compared to 53% for ambient experiments.

#### 3.1.2 Experiments at NOO_*x*-Pilot_ Setup

**Figure 6:**
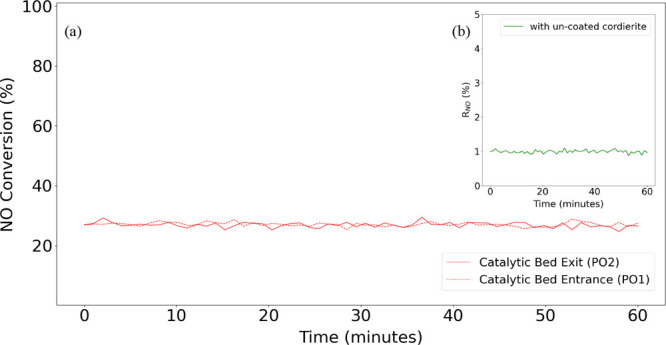
(a) Gas-phase NO to NO_2_ conversion (%) at the entrance and exit of bed with an uncoated cordierite monolith substrate as a function of time and (b) respective ratio (*R*_NO_) of conversion at the outlet to that at the entrance of bed with an uncoated cordierite monolith substrate as a function of time in the NOO_*x*-Pilot_ setup at 350 °C and 4 bar(g) pressure.

The gas-phase experiments at NOO_*x*-Pilot_ setup were measured using noncoated cordierite monoliths of the same dimensions as catalyst-coated monoliths. Figure 6 presents the gas phase NO conversion (%) measured with respect to the time at the PO1 sampling ports (at the entrance of the bed) and PO2 (at the exit of the bed) of the NOO_*x*-Pilot_ setup (presented in Figure S4). The ratio of conversion at the outlet to that at the entrance (*R*_NO_) is 1, indicating the absence of NO conversion through the noncoated cordierite monoliths (presented in (b) of Figure 6). However, even though the degree of oxidation is low between the inlet and outlet of a noncoated monolith, it is important to note that about 25% of NO is already oxidized before it reaches the catalyst bed.

Figure S6 presents meteorological data (precipitation and air temperature) collected during gas-phase measurements, along with the ammonia burner temperature of FAL-2, the gas temperatures through the bed in the NOO_*x*-Pilot_ setup at 350 °C and 4 bar(g) pressure. These measurements indicate that the inlet and outlet temperatures during the gas phase measurement remained quite similar to 1–2 °C drop through the bed. In addition, rain precipitation and the drop in air temperature during the time span of gas phase measurements did not have any major effect on NO conversion.

Figure S5 presents the results of the Aspen Plus simulation, where the homogeneous gas-phase NO conversion and temperature are calculated over the 10 cm bed. In summary, a 0.28% NO conversion and 0.5 °C increase in temperature were observed through a 10 cm catalytic bed. The simulation results are consistent with the NOO_*x*-Pilot_ gas-phase results presented in Figure 6.

### 3.2 Surface Characterization

Table 2 presents the surface area of the catalysts before and after metal impregnation, along with Ru metal dispersion. The total surface area was reduced with 5 wt % ruthenium impregnation. The surface area and ruthenium dispersion of the dry and wet-impregnated samples remained similar.

**Table 2:** N_2_ Physisorption Results Giving the BET Surface Area and Ru Dispersion from CO Chemisorption Measurements

catalyst	surfacearea^a^ [m^2^/g]	dispersion [%]	COuptake [μmol g^–1^]
γ-Al_2_O_3_	150		
Ru_Dry,γ–Al_2_O_3__	138	7%	6.1
Ru_Wet,γ–Al_2_O_3__	135	8%	6.8

a Average of two parallel experiments with the same material.

### 3.3 Effect of Precursor Metal Impregnation on Catalytic Activity

**Figure 7:**
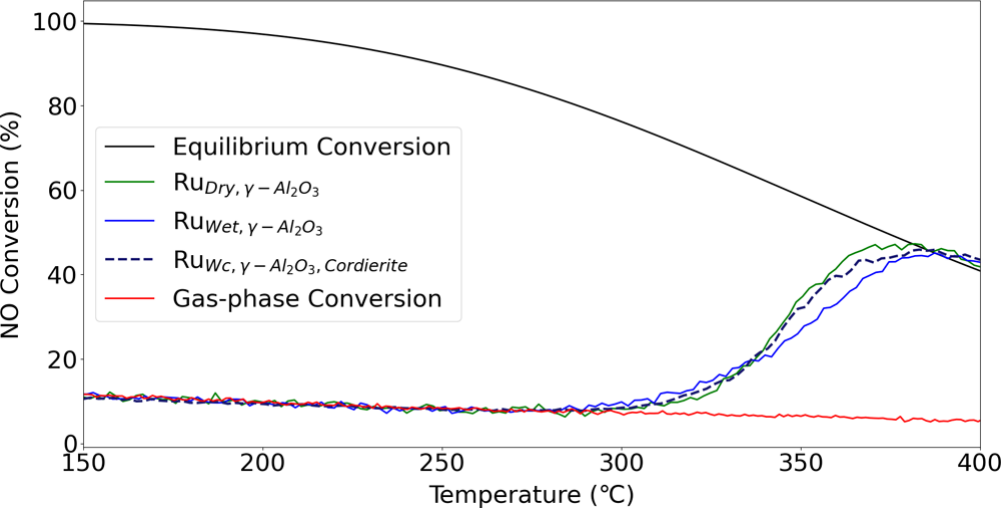
NO to NO_2_ conversion (%) as a function of temperature with feed (i): 10% NO, 6% O_2_, 15% H_2_O and rest Ar, heated at a rate of 5 °C/min at WHSV = 24,000 N cm^3^/g_gcat_ h and ambient pressure with Ru_Dry,γ–Al_2_O_3__, Ru_Wet,γ–Al_2_O_3__ and Ru_Wc,γ–Al_2_O_3_,Cordierite_ catalyst samples.

Figure 7 presents NO conversion for Ru_Dry,γ–Al_2_O_3__, Ru_Wet,γ–Al_2_O_3,__ and Ru_Wc,γ–Al_2_O_3_,Cordierite_ catalyst samples as a function of temperature with Feed (i): 10% NO, 6% O_2_, 15% H_2_O and balance Ar at 1 bar pressure. A simulated equilibrium curve (using the HSC Chemistry software^47^) and gas phase conversion are also presented in Figure 7 for comparison. The conversion of the gas phase from NO to NO_2_ was measured using 2.75 g of SiC mixed with 0.5 g of the γ-Al_2_O_3_ support, and the trend decreased with increasing temperatures, which confirms the inertness of the SiC and γ-Al_2_O_3_ support, reactor material, and the surfaces. The trend of gas-phase conversion also verifies the inverse Arrhenius behavior.^5^ The catalysts Ru_Dry,γ–Al_2_O_3__, Ru_Wet,γ–Al_2_O_3__ and Ru_Wc,γ–Al_2_O_3_,Cordierite_ were subjected to NO oxidation after a pretreatment with 5% H_2_/Ar at 400 °C. We found no notable differences between their NO oxidation activities, indicating that there is no direct relationship between the catalyst impregnation method and their respective NO oxidation activities.

### 3.4 Effect of Pressure on NO Oxidation Activity

**Figure 8:**
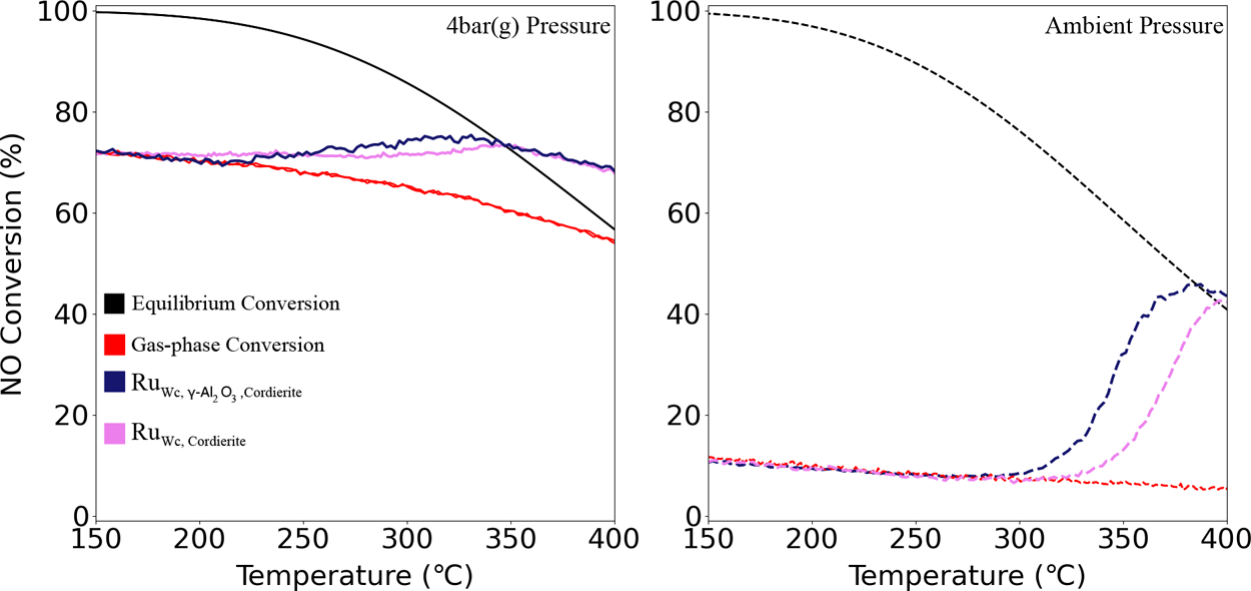
NO to NO_2_ conversion (%) as a function of temperature with feed (i): 10% NO, 6% O_2_, 15% H_2_O and rest Ar, heated at a rate of 5 °C/min at WHSV = 24,000 N cm^3^/g_gcat_ h with Ru_Wc,Cordierite_ and Ru_Wc,γ–Al_2_O_3_,Cordierite_ catalyst samples at ambient and 4 bar(g) pressure.

One other factor that bridges laboratory-scale activity testing with that of an industrial environment is pressure. To understand the effect of pressure on the coated monolith, a 5 wt % ruthenium was wash-coated on cordierite substrate directly and on alumina-coated cordierite. Figure 8 presents the conversion of NO to NO_2_ for the Ru_Wc,Cordierite_ and Ru_Wc,γ–Al_2_O_3_,Cordierite_ catalyst samples as a function of temperature with 10% NO, 6% O_2_, 15% H_2_O and rest Ar at ambient pressure and 4 bar(g).

Both samples were activated in 5% H_2_/Ar at 400 °C prior to activity testing. In Figure 8, the NO conversion (%) curves overshoots the equilibrium conversion at higher temperatures; this is due to the pressure difference in the NOO_*x*_ reactor outlet toward the FTIR gas analyzer (which measures the composition under ambient conditions).

The Ru_Wc,γ–Al_2_O_3_,Cordierite_ catalyst clearly outperformed Ru_Wc,Cordierite_ catalyst’s NO conversion at ambient pressures. However, at a pressure of 4 bar(g), both monoliths exhibited similar NO conversions of 72% at 350 °C, indicating no direct relation to the presence of the alumina wash coat and NO oxidation activity. This implies that even if the coating is imperfect, meaning that there is no alumina wash coat present in the coated monolithic catalyst, particularly within the channels, it would not lead to a decrease in the catalyst activity.

**Figure 9:**
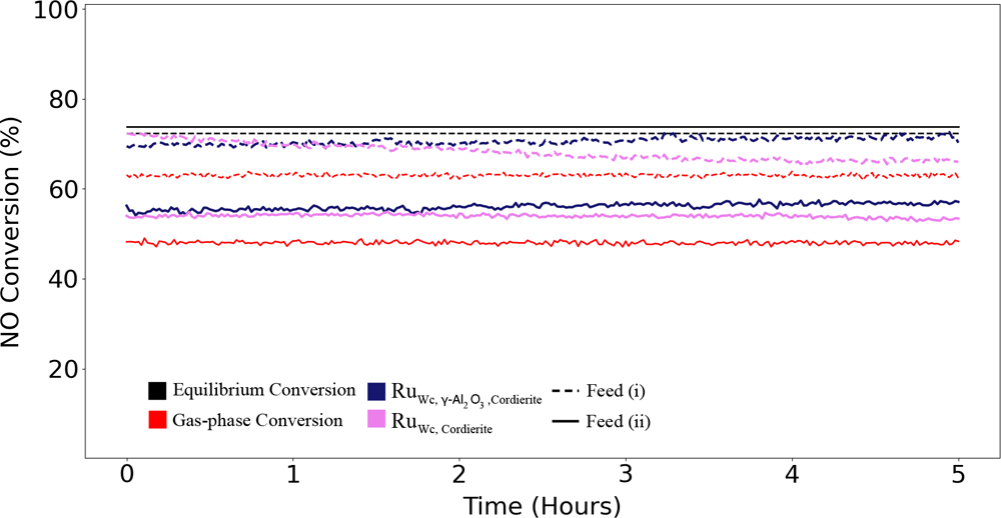
Isothermal experiment showing NO to NO_2_ conversion (%) as a function of time at 350 °C with feed (i): 10% NO, 6% O_2_, 15% H_2_O, and rest Ar and feed (ii): 8% NO, 2% NO_2_, 5% O_2_, 15% H_2_O and rest Ar, at WHSV = 24,000 N cm^3^/g_gcat_ h with Ru_Wc,Cordierite_ and Ru_Wc,γ–Al_2_O_3_,Cordierite_ catalyst samples at 4 bar(g) pressure.

Figure 9 presents isothermal conversion of NO to NO_2_ for 5 h at 350 °C with a feed of (i): 10% NO, 6% O_2_, 15% H_2_O and rest Ar and (ii): 8% NO, 2% NO_2_, 5% O_2_, 15% H_2_O and rest Ar for Ru_Wc,Cordierite_ and Ru_Wc,γ–Al_2_O_3_,Cordierite_ catalyst samples at 4 bar(g) pressure. The reason for conducting the isothermal tests at 350 °C was to have a significant catalytic conversion in addition to that of the gas phase conversion. In addition, the deactivation mechanisms are expected to accelerate at higher temperatures and closer to the equilibrium curve. In summary, the activity of Ru_Wc,Cordierite_ and Ru_Wc,γ–Al_2_O_3_,Cordierite_ catalyst samples was lower in feed ii than in feed (i). However, during 5 h, a decrease in activity was observed for the Ru_Wc,Cordierite_ monolith catalyst in the feed (i), and the activity of Ru_Wc,γ–Al_2_O_3_,Cordierite_ monolith catalyst increased. The latter phenomenon of increased activity of the Ru_Wc,γ–Al_2_O_3_,Cordierite_ monolith catalyst was also present in feed (ii). However, the activity of Ru_Wc,Cordierite_ monolith catalyst remained stable throughout the isothermal process in the feed (ii) with stable conversion of NO to NO_2_ of 56%. In both feeds (i) and ii, the Ru_Wc,γ–Al_2_O_3_,Cordierite_ monolith catalyst presented a conversion of 20% more than the gas phase conversion in the respective feeds. With clear activity differences between the Ru_Wc,γ–Al_2_O_3_,Cordierite_ and Ru_Wc,Cordierite_ catalyst samples, further studies were carried out on the effect of water and nitrogen dioxide on the Ru_Wc,γ–Al_2_O_3_,Cordierite_ catalyst.

### 3.5 Effect of Water and Nitrogen Dioxide on NO Oxidation Activity

From Figure 9, there is a clear decrease in the NO oxidation activity in the presence of nitrogen dioxide. This is due to the competitive adsorption of H_2_O and NO_2_ on the catalyst surface and a lower amount of O_2_ adsorption for NO conversion and also for NO_2_ evacuation.^22^ In the literature, nitrogen dioxide (NO_2_) and water (H_2_O) are well-known inhibitors of NO oxidation activity in various noble metals and metal oxides.^8,14,19,20,48,49,22^ Isothermal experiments were conducted at 350 °C and 4 bar(g) in the presence and absence of water and NO_2_ for 4 h to understand the effect of water and NO_2_ on the catalytic conversion of the Ru_Wc,γ–Al_2_O_3_,Cordierite_ catalyst sample.

**Figure 10:**
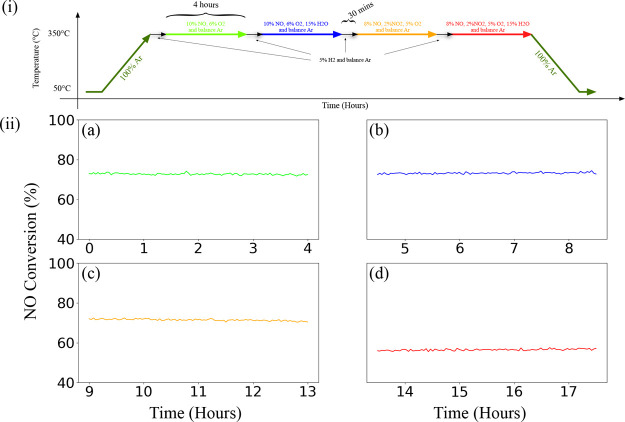
Effect of feed compositions on Ru_Wc,γ–Al_2_O_3_,Cordierite_ catalyst sample at 350 °C and 4 bar(g) pressure (i) program in NOO_*x*_ setup and (ii) respective NO to NO_2_ conversions (%) in NOO_*x*_ setup with respect to time in a feed of (a) 10% NO, 6% O_2_ and rest Ar, (b) 10% NO, 6% O_2_, 15% H_2_O, and rest Ar, (c) 8% NO, 2% NO_2_, 5% O_2_ and rest Ar and (d) 8% NO, 2% NO_2_, 5% O_2_, 15% H_2_O, and rest Ar at WHSV = 24,000 N cm^3^/g_gcat_ h.

**Figure 11:**
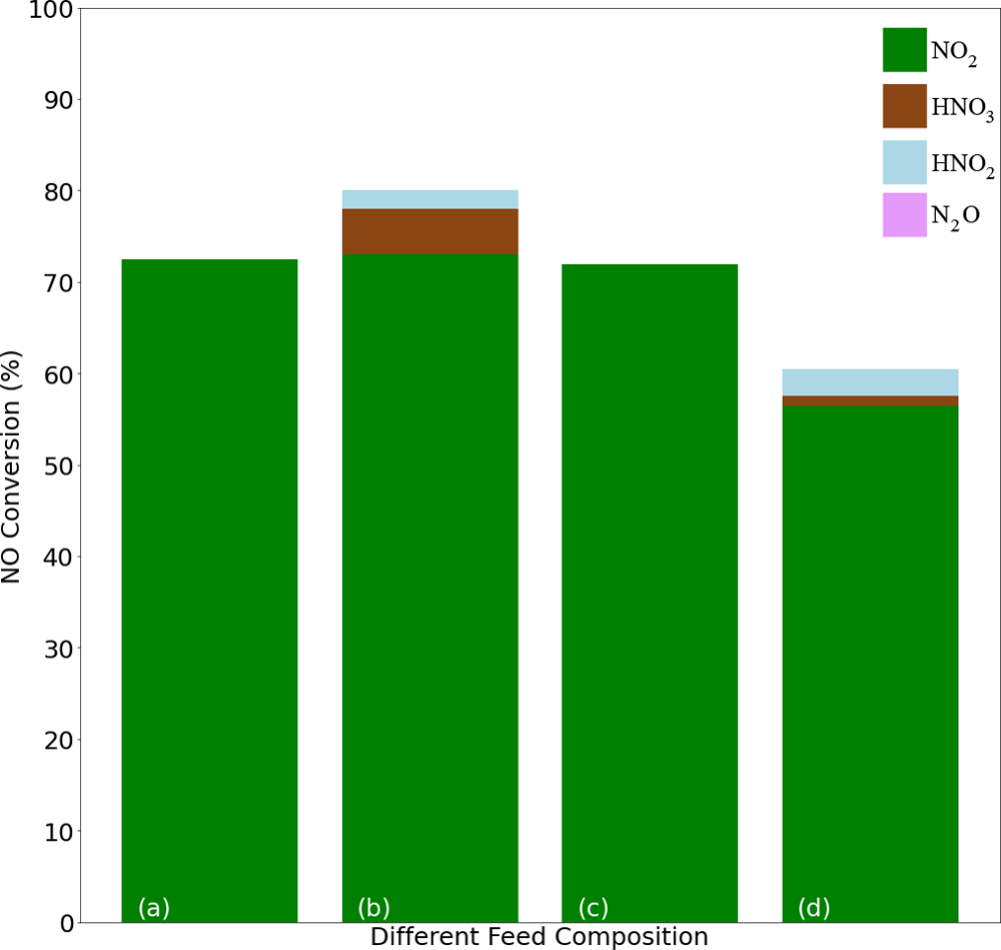
Average NO conversion (%) to NO_2_, HNO_3_, HNO_2_ and N_2_O during isothermal NO oxidation at 350 °C and 4 bar(g) pressure Ru_Wc,γ–Al_2_O_3_,Cordierite_ catalyst sample in a feed of (a) 10% NO, 6% O_2_ and rest Ar, (b) 10% NO, 6% O_2_, 15% H_2_O and rest Ar, (c) 8% NO, 2% NO_2_, 5% O_2_ and rest Ar and (d) 8% NO, 2% NO_2_, 5% O_2_, 15% H_2_O and rest Ar at WHSV = 24,000 N cm^3^/g_gcat_ h. NO to NO_2_ conversions (%) is presented in Figure 10.

Figure 10 presents conversions of isothermal NO to NO_2_ conversions at 350 °C and 4 bar(g) pressure for Ru_Wc,γ–Al_2_O_3_,Cordierite_ catalyst sample in a feed with 10% NO, 6% O_2_ and rest Ar, 10% NO, 6% O_2_, 15% H_2_O and rest Ar, 8% NO, 2% NO_2_, 5% O_2_ and rest Ar and 8% NO, 2% NO_2_, 5% O_2_, 15% H_2_O and rest Ar at WHSV = 24,000 N cm^3^/g_gcat_ h. Figure 11 presents the average NO conversion (%) to NO_2_, HNO_3_, HNO_2,_ and N_2_O during isothermal NO oxidation presented in Figure 10. The Ru_Wc,γ–Al_2_O_3_,Cordierite_ catalyst maintained an activity of 73–71% in the (a), (b), and (c) feed compositions, and a drop of 44% in conversion was observed when both H_2_O and NO_2_ were introduced into the feed (as presented in Figure 10). The formation of HNO_2_ and HNO_3_ is assumed to be a secondary reaction due to the reaction of NO_2_ and H_2_O (as presented in Figure S1). Therefore, the lower conversion of NO to NO_2_ in the feed (d) can be due to the presence of HNO_2_ in the feed, making the converted amounts of NO to NO_2_ difficult to estimate (as presented in Figure 11).

## 4 In-Situ and Ex-Situ XAS-XRD Characterization

**Figure 12:**
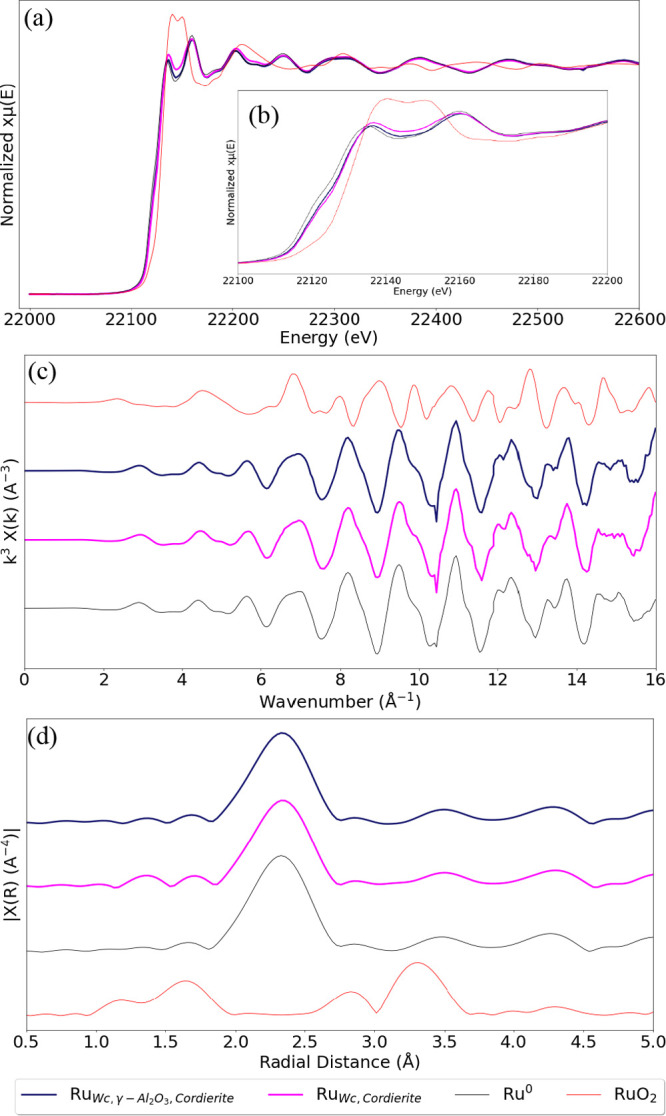
(a) Normalized EXAFS profiles at the Ru K edge, (b) normalized EXAFS profiles in the range of 22,100–22,200 eV, (c) EXAFS *k*-space (χ^3^) plots, and (d) EXAFS *R* space plots, collected in He atmosphere and 50 °C at the Ru K edge for spent catalyst samples of Ru_Wc,Cordierite_ and Ru_Wc,γ–Al_2_O_3_,Cordierite_ after 5 h isothermal NO oxidation in feed (i) and 4 bar(g) pressure (see Figure 9). The EXAFS profiles of the RuO_2_ and Ru^0^ standards are also plotted for comparison.

Spent catalyst samples of Ru_Wc,Cordierite_ and Ru_Wc,γ–Al_2_O_3_,Cordierite_ from 5 h isothermal NO oxidation in 10% NO, 6% O_2_, 15% H_2_O, and rest Ar were subjected to ex situ XAS measurements at BM31-SNBL, ESRF, France at Ru K edge. Figure 12 presents the normalized EXAFS, *R* space, and *k* space plots for the Ru^0^ foil, RuO_2_, and spent catalyst samples of Ru_Wc,Cordierite_ and Ru_Wc,γ–Al_2_O_3_,Cordierite_.

Comparing the EXAFS, *k*-space, and *R*-space profiles of the Ru^0^ and RuO_2_ standards with those of Ru_Wc,Cordierite_ and Ru_Wc,γ–Al_2_O_3_,Cordierite_, we observe that ruthenium is present as metallic in both catalyst samples.

Figure 12b closely presents the edge shift in the spent Ru_Wc,Cordierite_ and Ru_Wc,γ–Al_2_O_3_,Cordierite_ catalyst samples compared to the Ru^0^ foil, indicating oxidation. Drawing a comparison between the above findings and Figure 12b, we can safely dismiss the presence of any bulk RuO_2_ and deduce a possible surface oxidation, which is consistent with our previous results.^22^

**Figure 13:**
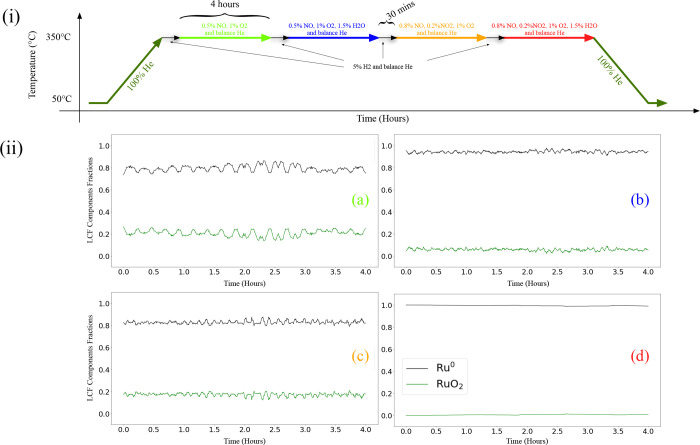
Effect of feed compositions on Ru_Wc,γ–Al_2_O_3_,Cordierite_ catalyst sample at 350 °C and 4 bar(g) pressure (i) program in in situ setup and (ii) respective in situ XANES-LCF fractions calculated (using Athena^43^) with respect to time (Reduced χ^2^ is between 1.8 and 2.1 × 10^–4^) in a feed of (a) 1% NO, 1% O_2_ and rest He, (b) 1% NO, 1% O_2_, 1.5% H_2_O and rest He, (c) 0.8% NO, 0.2% NO_2_, 1% O_2_ and rest He, and (d) 0.8% NO, 0.2% NO_2_, 1% O_2_, 1.5% H_2_O and rest He at WHSV = 24,000 N cm^3^/g_gcat_ h.

The Ru_Wc,γ–Al_2_O_3_,Cordierite_ catalyst sample was further investigated by using in situ XAS at BM31 to understand the effect of water and NO_2_ on ruthenium. Figure 13 (i) presents the in situ program and (ii) presents calculated in situ XANES-LCF fractions of RuO_2_ and Ru^0^ during the in situ program.

From the LCF results presented in Figure 13ii-a,c, in the absence of water, the Ru_Wc,γ–Al_2_O_3_,Cordierite_ catalyst oxidizes NO as a redox reaction with oscillating RuO_2_ and Ru^0^ fractions. In the presence of water (presented in Figure 13ii-b), the fraction of Ru^0^ increases, and a decrease in RuO_2_ fractions is observed with a lower oscillation amplitude between them. However, with both water and nitrogen oxide present in the system, no oscillations were found between the RuO_2_ and Ru^0^ fractions, and the Ru^0^ fraction was 1.

With Ru^0^ fraction as 1 and no indication of slight oxidation of ruthenium during NO oxidation in the presence of both water and nitrogen dioxide in the feed, one must reassess the role of HNO_2_ and HNO_3_. Both nitrous and nitric acid are well-known oxidizers. However, nitrous acid can act as a reducing agent by consuming oxygen to form nitric acid. We hypothesize that in Figure 13ii-d, with both water and nitrogen dioxide present in the feed, the Ru_Wc,γ–Al_2_O_3_,Cordierite_ catalyst is observed to be reduced due to HNO_2_ acting as a reducing agent.

### 4.1 Catalyst Activity in NOO_*x*-Pilot_ Setup

Pilot scale testing for Ru_Wc,γ–Al_2_O_3_,Cordierite_ catalysts was performed in the NOO_*x*-Pilot_ setup at Yara. When it comes to pilot-scale testing in the NOO_*x*-Pilot_ setup, new challenges arose. The testing was scheduled in the fall of 2023; however, due to technical constraints and unforeseen events, pilot testing commenced in early December 2023 (Winter). The weather was a hindrance to the pilot test, as the ideal location to test any catalyst to oxidize NO to NO_2_ would be downstream of the ammonia burner, which is typically cooled through heat exchangers in a nitric acid plant or cooled in ambient conditions with enough residence time.

Yara is a producer of nitrate fertilizers in Norway and a collaborator on this project. The scaled-up Ru_Wc,γ–Al_2_O_3_,Cordierite_ catalyst was tested in one of their pilot plant FAL-2 (Forso̷ks Anlegg). The typical flow in FAL-2 was 240–242 N m^3^/g_cat_ h, which is 10,000 times that in the NOO_*x*_ setup. Hence, going by space velocity, one would arrive at 10 kg of catalysts, which is ridiculously expensive in the context of ruthenium. As mentioned in the activity testing Section 2.3.2, we performed scale-up calculations based on the linear velocity of the NOO_*x*-Pilot_ to that of the NOO_*x*_ setup. However, the cost of the ruthenium precursor to match the linear velocity calculations was still higher, and a lower amount of catalyst (63 g) was used.

The catalyst was placed in a NOO_*x*-Pilot_ setup and continuously monitored for concentration differences (of NO, NO_2_, H_2_O, and NH_3_) and temperature differences (inlet and outlet of the bed). Furthermore, the weather data for the test area were also taken into account since the location of the NOO_*x*-Pilot_ setup was on the roof of the FAL-2 pilot building and exposed to ambient cooling. Activity tests were carried out for 65 h over 4 days, with measurements of 10 min in the first 2 days and continuous measurements on the third and fourth days using a TEMET FTIR analyzer. Short measurements on the first 2 days were due to technical difficulties in the FAL-2 pilot.

**Figure 14:**
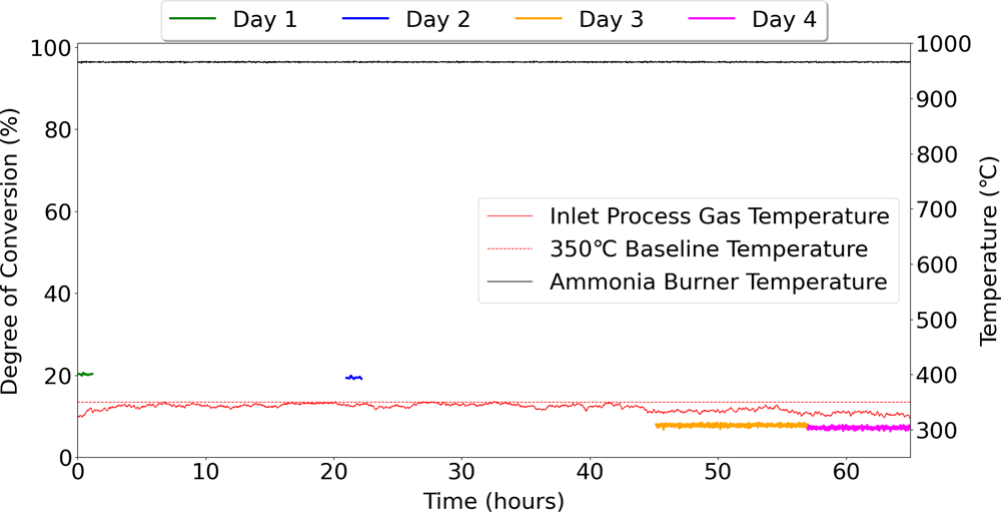
Degree of conversion (%) of Ru_Wc,γ–Al_2_O_3_,Cordierite_ catalyst as a function of time at NOO_*x*-Pilot_ setup with 240 N m^3^/g_cat_ h, 320–350 °C and 4 bar(g) pressure.

**Figure 15:**
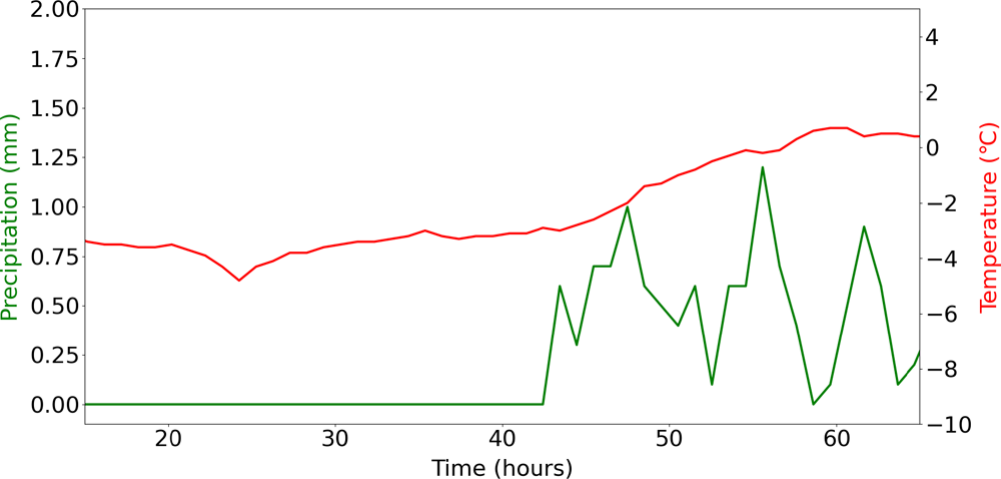
Average meteorological data on precipitation and air temperature over Porsgrunn-Hero̷ya (SN30256)^45^ as a function time during Ru_Wc,γ–Al_2_O_3_,Cordierite_ catalyst test at NOO_*x*-Pilot_ setup with 240 N m^3^/g_cat_ h, 320–350 °C and 4 bar(g) pressure.

Figure 14 presents the degree of conversion (%) measured over 65 h of pilot testing with the NOO_*x*-Pilot_ setup. Additionally, the figure also presents the gas temperature at the inlet of the catalyst bed and the ammonia burner temperatures of the FAL-2 pilot plant. The catalyst had 20% more oxidation than the gas phase and was stable until the inlet process gas temperature decreased after 40 h. Figure 15 presents the precipitation and air temperature data around the FAL-2 pilot plant for the duration of the catalyst test presented in Figure 14. The figure clearly indicates the increase in air temperature and precipitation after 40 h, which explains the drop in gas temperature at the entrance of the catalytic bed and the decrease in catalyst activity presented in Figure 14.

### 4.2 Ex-Situ XRD Characterization

**Figure 16:**
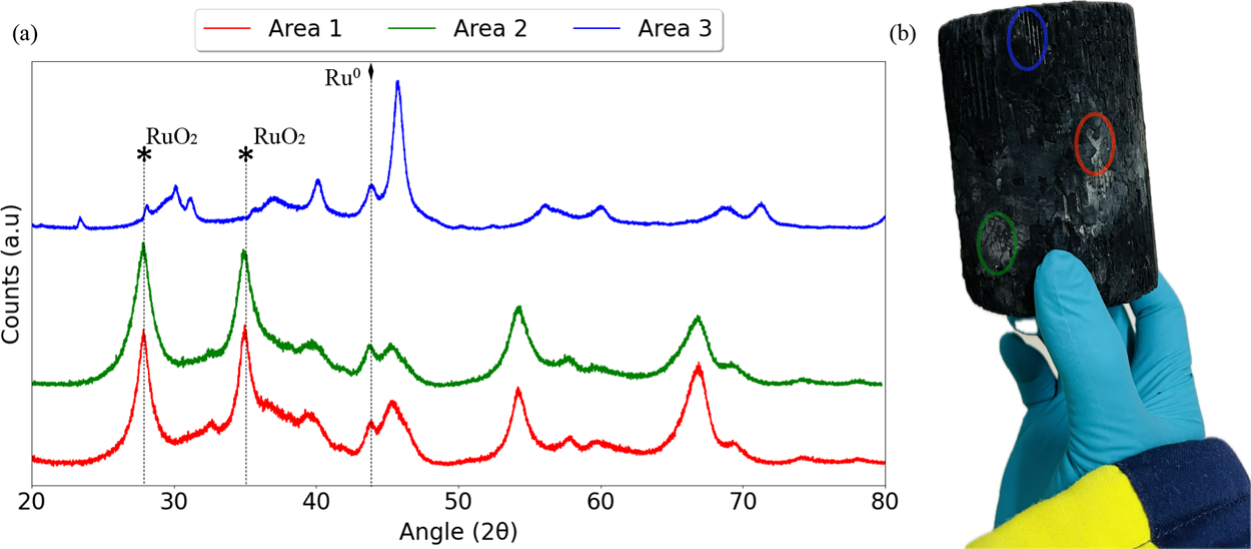
(a) X-ray diffractograms of three marked areas (1, 2, and 3) of spent Ru_Wc,γ–Al_2_O_3_,Cordierite_ catalyst after pilot testing at the NOO_*x*-Pilot_ setup with 240 N m^3^/g_cat_ h, 320–350 °C and 4 bar(g) pressure with diffraction peaks of RuO_2_ (PDF-04–003–2008) are represented as * and Ru^0^ (PDF-00–006–0663) are represented as ⧫ respectively. (b) Presents an image of the spent Ru_Wc,γ–Al_2_O_3_,Cordierite_ catalyst after pilot testing at the NOO_*x*-Pilot_ setup with 240 N m^3^/g_cat_ h, 320–350 °C and 4 bar(g) pressure, marking the three distinctly colored areas.

The spent characterizations of the Ru_Wc,γ–Al_2_O_3_,Cordierite_ catalyst proved to be challenging. The monolith (presented in Figure 16b) was too large to be used in any known characterization technique, and parts of the spent monoliths easily crumbled and disintegrated into flakes. Therefore, the cohesiveness of the wash coat and cordierite was greatly affected by the pilot tests.

Two areas of the monoliths were very peculiar, as they were distinctly colored from the third area (which appeared pretty much black). Samples were obtained from each of these areas by scraping that specific region. X-ray diffractograms of the three marked areas on the spent Ru_Wc,γ–Al_2_O_3_,Cordierite_ catalyst were collected and are presented in Figure 16a. The diffractograms presented in Figure 16a reveal that the catalyst is more oxidized in the red area than in the blue area. From the LCF results presented in Figure 13 (ii), the catalyst was partially oxidized or fully reduced during 4 h of NO oxidation. Therefore, we believe that the oxidation observed in the red and green areas is due to the formation of nitric acid at low temperatures during the start of the FAL-2 pilot plant (Section 2.3.2).

Figure 16, area blue diffractograms, presents peaks corresponding to Ru^0^ and cordierite. However, peaks corresponding to wash-coated γ-Al_2_O_3_ were absent, indicating that ruthenium was directly wash-coated on cordierite. It is very unlikely that even trace amounts of alumina were not present in the wash coat (blue area). Therefore, we believe that the ruthenium-alumina wash coat must have been lost as flakes during the pilot tests and that what is left behind is the ruthenium wash coat on the cordierite.

## 5 Conclusions

In this work, we explored the NO to NO_2_ oxidation capacity of ruthenium and the γ-Al_2_O_3_ support wash coated on a cordierite monolith. The effect of precursor impregnation was studied; along with pressure and inhibition effects of H_2_O and the product NO_2_ in the feed. The catalytic conversion trends of wash-coated monoliths were similar to those of powder catalyst, implying that transitioning to a monolith reactor had a minimal impact on the catalytic activity of ruthenium. The effects of pressure on wash-coated monoliths were studied, and both ruthenium- and ruthenium-alumina wash-coated monoliths exhibited similar activity. This implied that an imperfect coating would not lead to a decrease in the catalyst activity.

Upon examination of the effects of water and nitrogen dioxide on ruthenium-alumina-wash coated monoliths, the catalyst maintained a conversion of 73–71% in the absence of water and nitrogen dioxide and in the presence of water or nitrogen dioxide. However, when both water and nitrogen dioxide were introduced into the feed, the conversion from NO to NO_2_ decreased by 44% due to the presence of HNO_2_ in the feed.

The in situ XAS revealed that in the absence of water and/or NO_2_, the Ru_Wc,γ–Al_2_O_3_,Cordierite_ catalyst oxidizes NO as a redox reaction with oscillating fractions of RuO_2_ and Ru^0^. The Ru_Wc,γ–Al_2_O_3_,Cordierite_ catalyst maintained a steady degree of conversion of NO to NO_2_ for 65 h in the pilot tests conducted at Yara, a Norwegian fertilizer company. The results presented in this work clearly provide evidence to support the idea that ruthenium on a gamma-alumina catalyst support can oxidize NO to NO_2_ under industrial nitric acid production conditions. With the activity demonstration of the Ru_Wc,γ–Al_2_O_3_,Cordierite_ catalyst in the NOO_*x*-Pilot_ setup at Yara, it marks an important step in the intensification of the Ostwald process.

## Supporting Information Available

Program simulating different feed compositions and their corresponding average conversions, details of experimental lab setups, in situ XAS-XRD setups, NOO_*x*-Pilot_ experimental setups, results of simulated gas-phase NO oxidation per NOO_*x*-Pilot_ reactor length, and weather data during catalyst testing in NOO_*x*-Pilot_ setup (PDF)
