# Supplementary material for: Ostwald Process Intensification by Catalytic Oxidation of Nitric Oxide
Source: ACS Omega. 2025 Jan 9;10(2):2197–211. doi: 10.1021/acsomega.4c09111 (PMC11755144; doi:10.1021/acsomega.4c09111)
Supplement: Supplementary file 1 — ao4c09111_si_001.pdf [file ao4c09111_si_001.pdf]

Supplementary Information (SI)

Ostwald Process Intensification by Catalytic Oxidation of Nitric Oxide

Jithin Gopakumar[1], Rune Myrstad[2], Rebecka Børresen Anda[1], Halvor Øien[3], Bjørn Christian Enger[2], David Waller[3], Magnus Rønning[1]*

1.Norwegian University of Science and Technology (NTNU), Department of Chemical Engineering, Sem Sælands vei 4, NO-7491 Trondheim, Norway

2.SINTEF Industry, Kinetic, and Catalysis group, P.O. Box 4760 Torgarden, NO-7465 Trondheim, Norway

3.YARA Technology Center, Herøya Forskningspark, Bygg 92, Hydrovegen 67, NO-3936 Porsgrunn, Norway

**Corresponding author, E-mail address: magnus.ronning@ntnu.no (M. Rønning)*

S1 Programme Simulating Different Feed Compositions

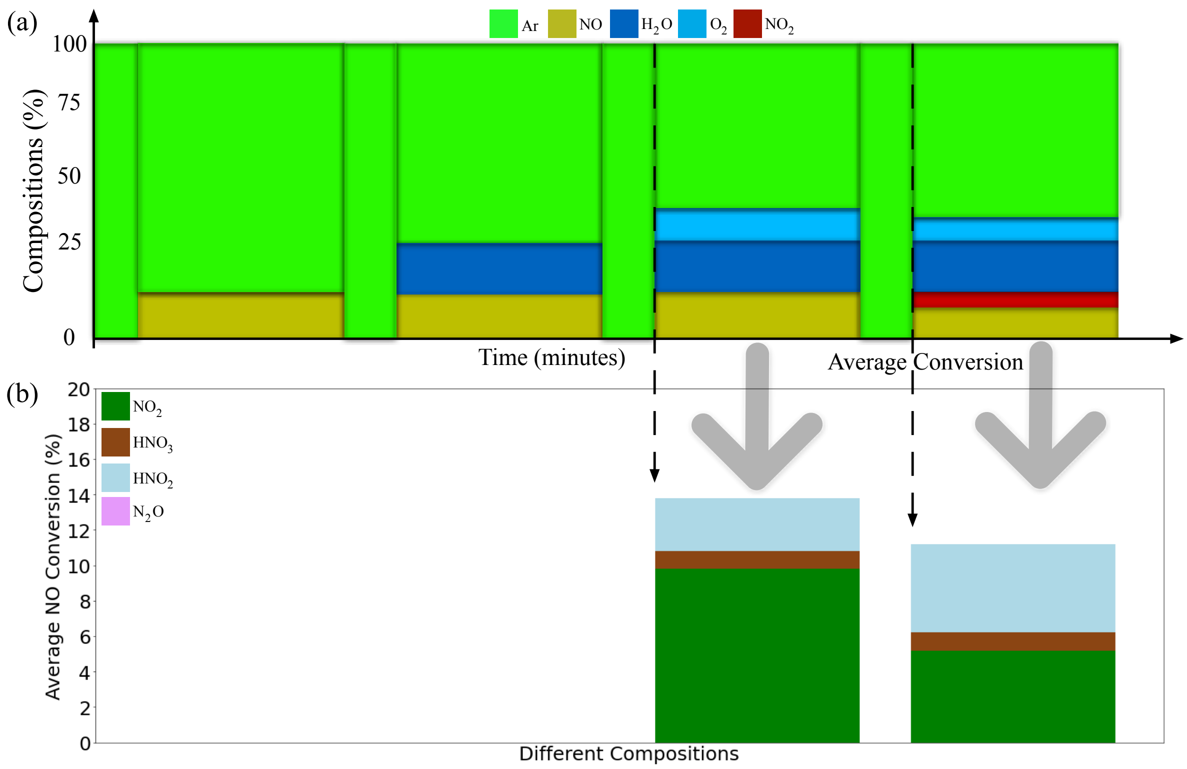

Figure S1: (a) Programme simulating different feed compositions using Ar, NO, NO_2, O_2 and H_2O. (b) Average conversion of NO to NO_2, HNO_3, HNO_2 and N_2O at 200 Ncm^3/min, 350°C and ambient pressure while simulating different feed compositions presented in (a).

Fig. S1 presents the average NO conversion with different feed compositions in an empty reactor at 350°C and ambient pressure. The results also reveal the absence of HNO_3 and HNO_2 when NO and steam are fed into the reactor. However, the conversion of NO to NO_2, HNO_3 and HNO_2 occurs when oxygen is fed into the system, a clear indication that secondary reactions are responsible for the formation of HNO_3 and HNO_2. No increase in conversion towards HNO_3 were found with addition of NO_2 to the system, while an increase in conversion to HNO_2 was observed.

S2 Details of Experimental Lab Setup

A SS-316 tubular reactor of 9.7 mm inner diameter was used for the activity testing. For heating the reactor, a heat block is used with four cartridge heaters controlled by a Eurotherm. Two thermocouples, TT_1 and TT_2 (as presented in Fig. 1) are placed in the catalyst bed and heat block, respectively, for precise control of the temperature by Eurotherm (presented in Fig. 1. The temperature control profile for the catalyst bed and the respective confidence interval fit during isothermal operation are presented in our previous work[22]. Due to the gas phase oxidation of NO, a tube-in-tube design (presented in Fig. 1) was used to dose NO, NO_2, H_2O and O_2, so that they come in contact at the mouth of the reactor. To minimise the gas-phase conversion after the reactor and to protect the MKS gold mirror from corrosive gas, the product gases were diluted using argon with a dilution ratio of 5-10 (Argon flow/Product gas flow). The measurements of thermocouples (TT_1 and TT_2), FTIR, and mass spectrometer are all in real time with a precision of milliseconds. Furthermore, the time gap between the FTIR and the mass spectrometer is corrected by pulsing 5% NO_2 in inert.

S3 Details of *In-situ* XAS-XRD Experimental Setup

Fig. S2 presents an experimental setup used for *in-situ* XAS-XRD experiments at SNBL, ESRF, France. The setup includes a dedicated setup with mass, pressure and/or liquid flow controllers used to feed desired concentrations of NO, NO_2 (when present), O_2, H_2O and He (WHSV: 24,000 $Ncm^3/g_{cat}.h$ at ambient or 4 bar(g) pressure. Automated switch valves were used to control the *in-situ* rig from the control room at SNBL. A custom LabVIEW program was used to control the rig.

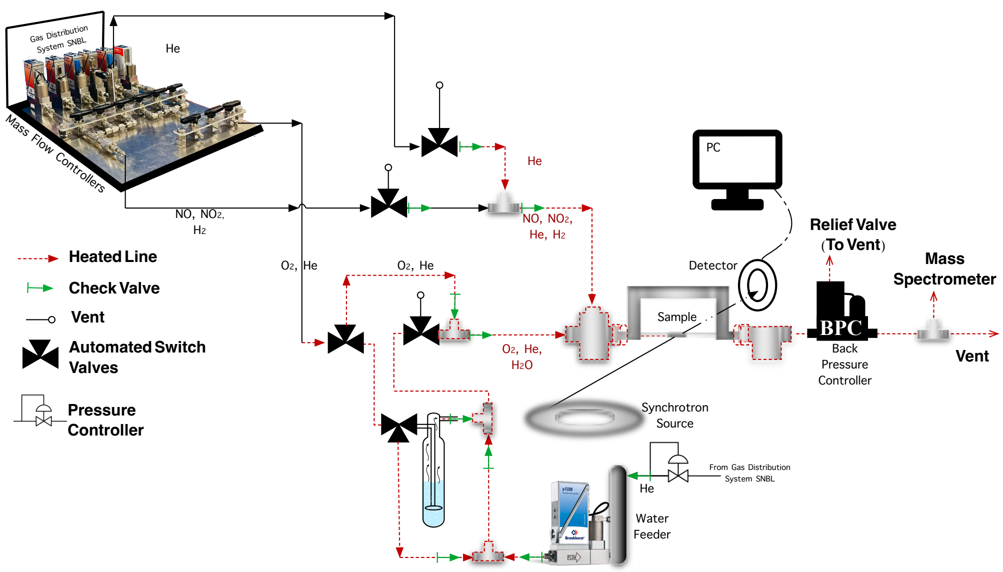

Figure S2: *In-situ* XAS-XRD experimental setup for nitric oxide oxidation experiments at SNBL, ESRF, France.

S4 Details of $NOO_{x-Pilot}$ Experimental Setup

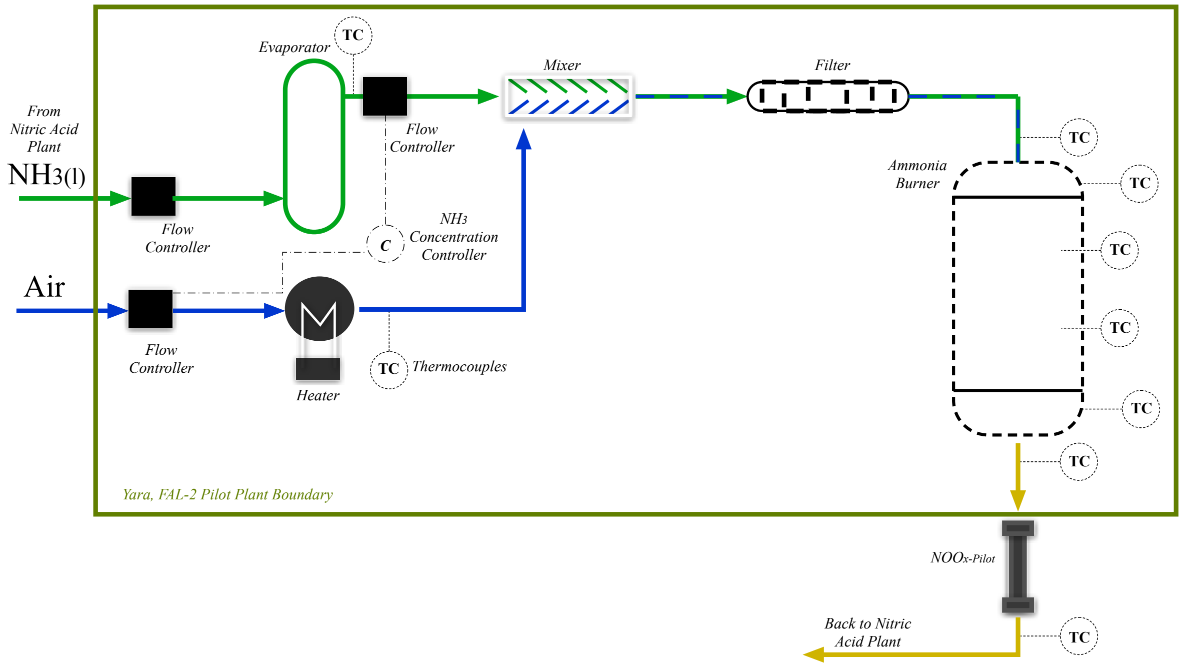

Figure S3: Schematic representation of FAL-2 pilot at Yara, Porsgrunn, Norway.

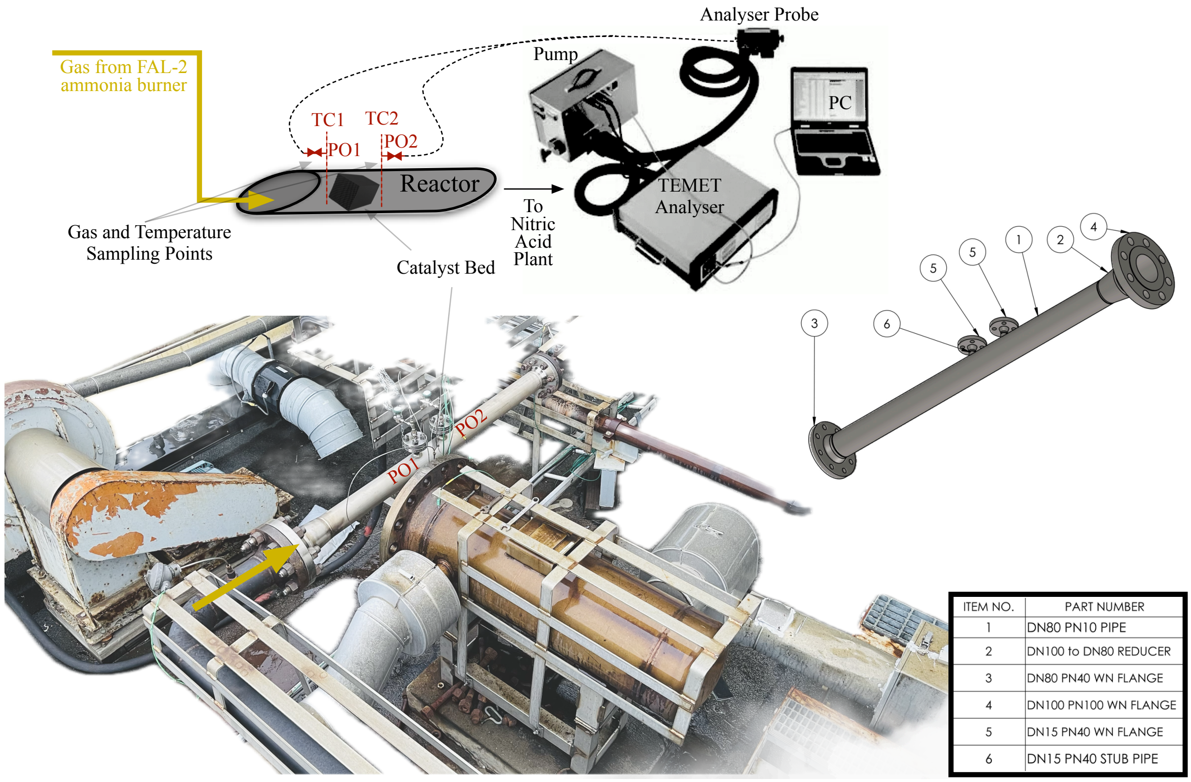

Figure S4: $NOO_{x-Pilot}$ experimental setup for nitric oxide oxidation experiments located downstream of FAL-2 pilot ammonia burner at Yara, Porsgrunn, Norway.

S5 Simulated Gas-Phase NO Oxidation per $NOO_{x-Pilot}$ Setup Reactor Length

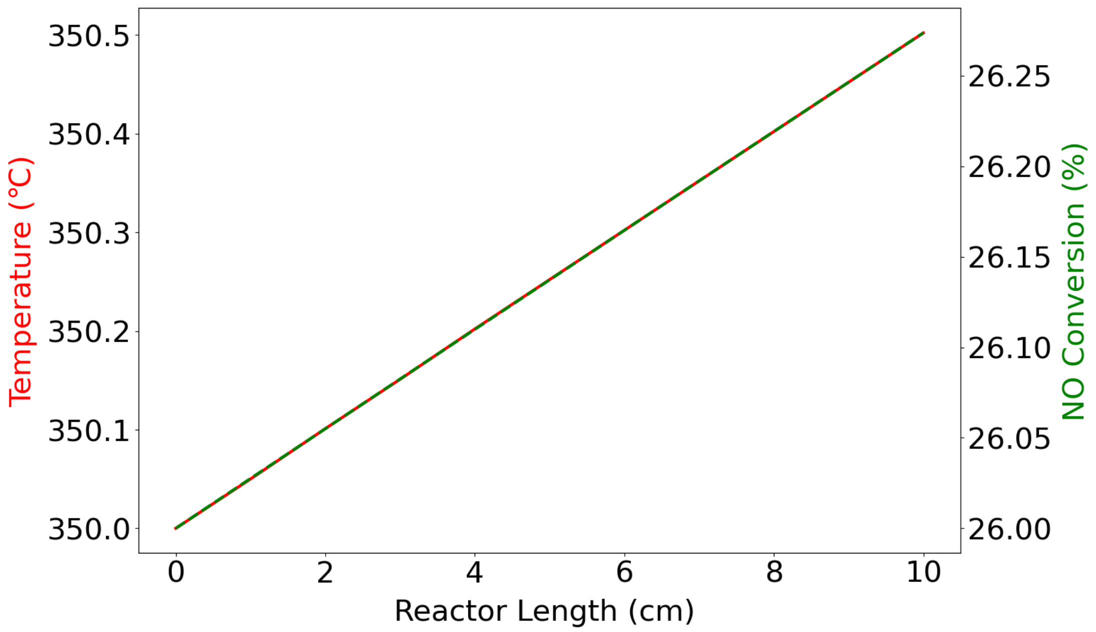

Figure S5: Aspen Plus simulated homogeneous gas-phase NO oxidation through 10 cm catalytic bed with 350°C inlet temperature, 4bar(g) pressure and 240 Nm^3/h flow.

S6 Weather Data

Acquired weather data during gas-phase experiments in $NOO_{x-Pilot}$ setup with uncoated cordierite monolith.

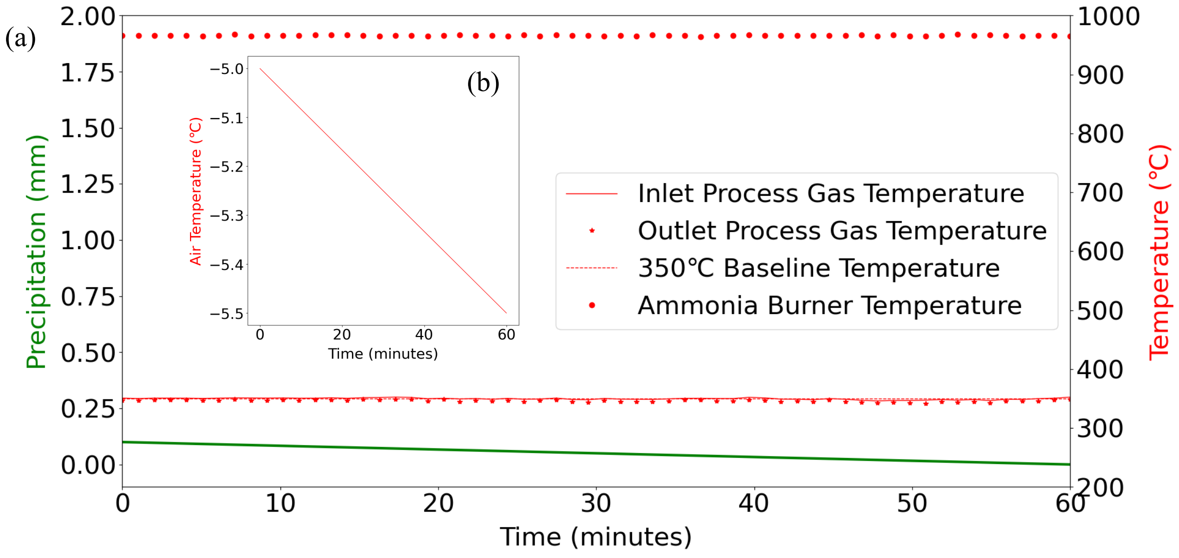

Figure S6: (a) Precipitation data, ammonia burner temperature, temperature of inlet and outlet process gas and (b) air temperature as a function of time during the gas-phase NO conversion measurements in the $NOO_{x-Pilot}$ setup at 350°C and 4 bar(g) pressure.
